# Genome-wide co-localization of Polycomb orthologs and their effects on gene expression in human fibroblasts

**DOI:** 10.1186/gb-2014-15-2-r23

**Published:** 2014-02-03

**Authors:** Helen Pemberton, Emma Anderton, Harshil Patel, Sharon Brookes, Hollie Chandler, Richard Palermo, Julie Stock, Marc Rodriguez-Niedenführ, Tomas Racek, Lucas de Breed, Aengus Stewart, Nik Matthews, Gordon Peters

**Affiliations:** 1 Molecular Oncology Laboratory, CRUK London Research Institute, 44 Lincoln’s Inn Fields, London WC2A 3LY, UK; 2 Bioinformatics and Biostatistics Service, CRUK London Research Institute, 44 Lincoln’s Inn Fields, London WC2A 3LY, UK; 3 Advanced Sequencing Facility, CRUK London Research Institute, 44 Lincoln’s Inn Fields, London WC2A 3LY, UK; 4 Present address: Gene Function Laboratory, Breakthrough Breast Cancer, Institute of Cancer Research, 237 Fulham Road, London SW3 6JB, UK; 5 Present address: Cancer Research Technology, Wolfson Institute for Biomedical Research, The Cruciform Building, Gower Street, London WC1E 6BT, UK; 6 Present address: Departamento de Anatomía y Embriología Humana I, Complutense University of Madrid, Madrid, Spain; 7 Present address: Dr von Haunersches Kinderspital, Lindwurmstr 2a, 80337, Munich, Germany; 8 Present address: Graduate School of Business, Stanford University, Stanford, CA 94305-7298, USA

## Abstract

**Background:**

Polycomb group proteins form multicomponent complexes that are important for establishing lineage-specific patterns of gene expression. Mammalian cells encode multiple permutations of the prototypic Polycomb repressive complex 1 (PRC1) with little evidence for functional specialization. An aim of this study is to determine whether the multiple orthologs that are co-expressed in human fibroblasts act on different target genes and whether their genomic location changes during cellular senescence.

**Results:**

Deep sequencing of chromatin immunoprecipitated with antibodies against CBX6, CBX7, CBX8, RING1 and RING2 reveals that the orthologs co-localize at multiple sites. PCR-based validation at representative loci suggests that a further six PRC1 proteins have similar binding patterns. Importantly, sequential chromatin immunoprecipitation with antibodies against different orthologs implies that multiple variants of PRC1 associate with the same DNA. At many loci, the binding profiles have a distinctive architecture that is preserved in two different types of fibroblast. Conversely, there are several hundred loci at which PRC1 binding is cell type-specific and, contrary to expectations, the presence of PRC1 does not necessarily equate with transcriptional silencing. Interestingly, the PRC1 binding profiles are preserved in senescent cells despite changes in gene expression.

**Conclusions:**

The multiple permutations of PRC1 in human fibroblasts congregate at common rather than specific sites in the genome and with overlapping but distinctive binding profiles in different fibroblasts. The data imply that the effects of PRC1 complexes on gene expression are more subtle than simply repressing the loci at which they bind.

## Background

Polycomb-group (PcG) proteins were originally identified by their developmental effects in *Drosophila* but their mammalian counterparts are implicated in a wide variety of physiological processes, including pluripotency, imprinting, differentiation and senescence [1-3]. Senescence is a state of permanent cell cycle arrest that occurs in response to various forms of cellular stress and acts as a front-line defense against potentially oncogenic lesions [4,5]. Genetic ablation of a number of PcG genes results in premature senescence, in part because of de-repression of the *CDKN2A* tumor suppressor [6,7].

PcG proteins operate within multi-component complexes, the best characterized of which are termed Polycomb repressive complexes 1 and 2 (PRC1 and PRC2) [1-3]. PRC2 is responsible for tri-methylating histone H3 on lysine 27 (H3K27me3), generally considered to be a mark of transcriptional repression, and apart from duplication of the Ezh subunit, the core components of the complex are conserved between flies and mammals [8].

In contrast, the PRC1 components have undergone considerable expansion during evolution. The prototypic PRC1 complex in *Drosophila* comprises equimolar amounts of Polycomb (Pc), Posterior sex combs (Psc), Polyhomeotic (Ph) and Sex combs extra (Sce) [9,10]. However, as human cells encode five Pc, six Psc, three Ph and two Sce orthologs, there are multiple variants of PRC1 [2,8,11]. Biochemical analyses indicate that a typical PRC1 complex contains a single representative from each gene family [7,12-15] and, although it has not been formally shown that all of the orthologs participate in functional PRC1-type complexes, there could in principle be up to 180 different permutations. The situation is further complicated by the ability of the Psc and Sce subunits to form functional complexes that contain RYBP or YAF2 rather than the Pc and Ph orthologs [15-17].

The Psc and Sce subunits combine to form an E3 ubiquitin ligase that mono-ubiquitinates histone H2A on lysine 119 (H2AK119), a modification that is also associated with transcriptional repression [1-3]. However, it has not been conclusively established whether the H2AK119 modification is essential for blocking transcription and whether it is carried out by the prototypic PRC1 or alternative complexes [15-20].

The reason for the expansion of PRC1 families is unclear as there is currently little evidence for functional diversification [15,21-23]. It has been generally assumed that particular family members operate in different cell lineages or that the various permutations of PRC1 regulate specific sets of target genes [15,24]. For example, CBX7 is the predominant Pc ortholog in embryonic stem (ES) cells but its expression declines during differentiation and it is replaced by CBX2, CBX4 and CBX8 [24,25]. If these proteins bind selectively, as suggested, then the number of PcG target genes would potentially increase in cell types that express multiple variants of the canonical PRC1 complex.

Here we investigate the genome-wide distribution of the PRC1 components expressed in two strains of normal human fibroblasts (HFs), a somatic cell type required for the physical integrity of multiple tissues and the classical model for the study of cellular senescence. Contrary to expectations, we find that three Pc orthologs (CBX6, 7 and 8) and both Sce proteins (RING1 and RING2) have very similar binding patterns throughout the genome with little evidence for target gene specificity. Moreover, by assembling a panel of antibodies that support chromatin immunoprecipitation (ChIP) of the endogenous proteins, we find that 11 of the PRC1 core components expressed in HFs display virtually identical binding patterns at representative loci. Importantly, sequential ChIP with antibodies against different members of the Pc, Ph and Sce families implies that multiple variants of PRC1 associate simultaneously with the same chromatin. At many loci, the binding patterns have an inherent architecture that is faithfully conserved between the two strains of HF but at an equivalent number of loci the binding is strain specific. Surprisingly, there is no strict correlation between the presence and position of the PRC1 peaks and RNA expression from adjacent gene(s). Moreover, the genomic location of the Pc orthologs remains unchanged in senescent HFs. The findings are consistent with the idea that mammalian PRC1 proteins congregate as complexes of complexes and that their control over gene expression is more subtle than simply blocking transcription of the genes to which they bind.

## Results

### Expression of PRC1 genes in human fibroblasts

**Figure 1:**
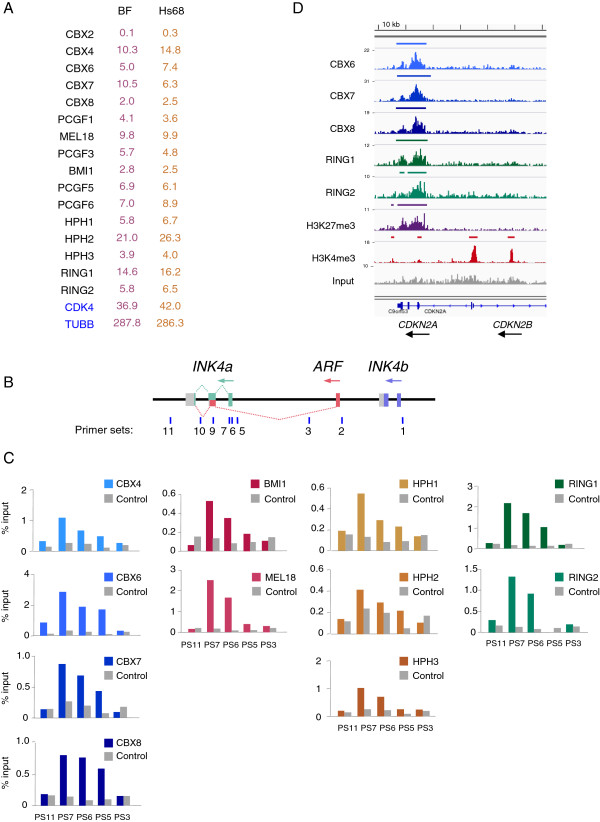
**Multiple PRC1 components have similar binding profiles at the *CDKN2A *locus. (A)** Relative expression levels of the core PRC1 components in the BF and Hs68 strains of primary fibroblasts. The numbers are mean FPKM values from four independent RNA-sequencing analyses. *CDK4* and *TUBB* are shown as controls. **(B)** Genomic organization of the human *CDKN2A/B* locus with colored boxes depicting the exons that encode p16^INK4a^, p14^ARF^ and p15^INK4b^. The locations of primer sets used for ChIP analyses are shown below, numbered according to Bracken *et al*. [27]. The sequences are described in Additional file 6: Table S3. **(C)** Examples of ChIP data using antibodies against four Pc proteins (blue), two Psc proteins (pink), three Ph orthologs (orange) and both Sce proteins (green) using the indicated primer sets. Binding is shown as the percentage of input. Non-specific antibodies or pre-immune serum were used as controls as appropriate (shown in grey). **(D)** Screenshot showing DNA sequence tag densities at the *CDKN2*A locus following ChIP-sequencing with the indicated antibodies in BF fibroblasts. The maximum coverage for each track is shown on the left and a size bar is included above the IGV screenshot. ChIP, chromatin immunoprecipitation; kb, kilobase; FPKM, fragments per kilobase of gene per million fragments mapped; IGV, Integrative Genomics Viewer.

Most studies of mammalian PcG proteins and their potential target genes have focused on ES cells and *in vitro* models of differentiation. We chose to investigate the situation in HFs, in part because of the evidence linking PRC1 proteins with senescence, via effects on the *CDKN2A* locus [26], but also because HFs express a much broader repertoire of PRC1 genes than ES cells. For example, deep sequencing of RNA isolated from the BF strain of adult breast fibroblasts and the Hs68 strain of neonatal foreskin fibroblasts suggested that 15 of the 16 genes encoding core PRC1 components are expressed, albeit at variable levels (Figure 1A). Among the four gene families, the highest expression was observed for CBX4, MEL18, HPH2 and RING1. In contrast to mouse embryo fibroblasts [24,25], the HFs expressed substantial amounts of CBX7 whereas CBX2 was barely detectable at the RNA level.

### Multiple PRC1 complexes bind simultaneously at the *CDKN2A* locus

The RNA expression data implied that HFs have the potential to express many different permutations of PRC1 and are therefore an ideal system in which to investigate target specificity. Using the *CDKN2A* locus as a test bed for antibody evaluation, we assembled a panel of custom-made and commercially available reagents that support ChIP of endogenous PRC1 components in primary HFs. A number of PRC1 components have been previously located at *CDKN2A* using a validated set of PCR primers (Figure 1B and [7,12,27]) but our survey has added substantially to the list of proteins involved. We found that four Pc proteins (CBX4, 6, 7 and 8), two Psc proteins (BMI1 and MEL18), all three Ph orthologs (HPH1, 2 and 3) and both Sce proteins (RING1 and 2) were not only enriched at the *CDKN2A* locus but had virtually identical binding profiles (Figure 1C). Importantly, we confirmed that the antibodies did not cross-react with members of the same family, even when they were over-expressed (Additional file 1: Figure S1).

### Genome-wide profiling of PRC1 binding sites

To determine whether PRC1 proteins are co-localized at other target genes, we conducted genome-wide ChIP-sequencing (ChIP-seq) with antibodies against three of the Pc orthologs and both Sce orthologs for the BF and Hs68 fibroblasts. We reasoned that by comparing the binding profiles of CBX6, CBX7 and CBX8, we could ascertain whether they regulate distinct or overlapping sets of target genes in HFs. Conversely, as every catalytically active PRC1 complex should contain either RING1 or RING2, the combined dataset should provide an impression of all PRC1 binding sites in the genome, which would complement the CBX profiles. For reference purposes, we also generated ChIP-seq profiles for H3K27me3 and H3K4me3 for the same cells. *CDKN2A* is shown as an example in Figure 1D. Sequencing was performed on the Illumina GAIIx platform and typically generated between 12 and 60 million 36-bp reads that could be mapped to the hg19 release of the human genome. The raw and processed data have been deposited under GEO accession number [GEO:GSE40740] [28].

**Figure 2:**
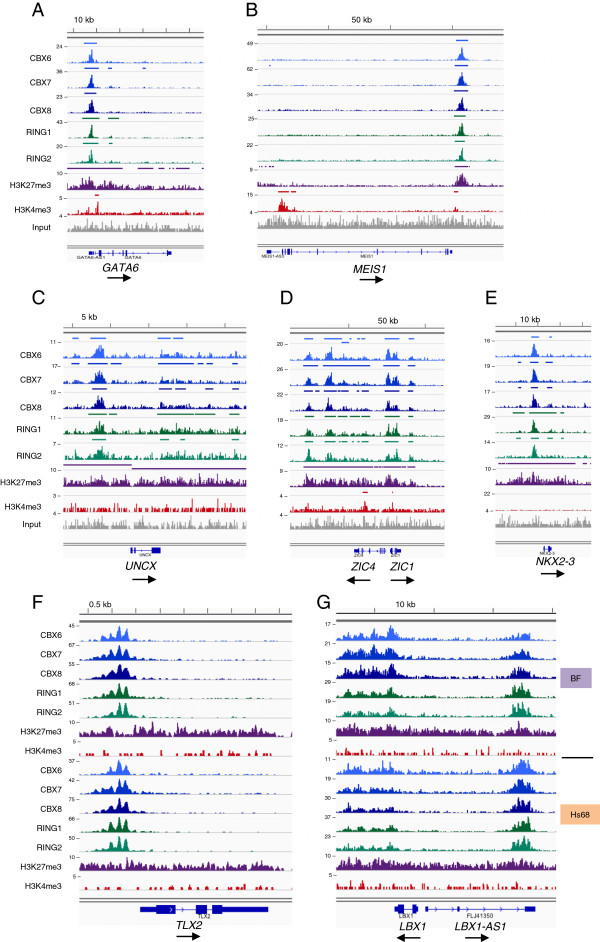
**Representative examples of ChIP-sequencing data at annotated loci.** The panels show DNA sequence tag densities observed following ChIP-sequencing with the indicated antibodies at *GATA6***(A)**, *MEIS1***(B)**, *UNCX***(C)**, *ZIC4***(D)** and *NKX2-3***(E)**, in the BF strain of HFs. Panels **(F)** and **(G)** show the equivalent ChIP-sequencing data for the *TLX2* and *LBX1* loci in both BF and Hs68 cells. The maximum coverage for each track is shown on the left and a size bar is included above the IGV screenshot. The genomic organization of each locus according to the UCSC genome browser is shown below, with arrows indicating the direction of transcription. HF, human fibroblast; kb, kilobase; IGV, Integrative Genomics Viewer.

Unlike the inferences drawn from other cell systems [15,24], the five PRC1 proteins showed remarkably similar binding patterns at multiple sites throughout the genome (Figure 2). However, the bioinformatic interpretation was complicated by the fact that peak-calling algorithms, such as MACS [29], often identified different numbers of peaks at particular loci depending on the density of the sequence reads for each antibody. To try to clarify the situation, the ChIP-seq was repeated with chromatin prepared from independent cell populations rather than simply increasing the number of reads. Merging of replicate datasets improved the definition of coincident binding sites but did not completely resolve the issue of peak calling. For example, it was common to find regions where a robust peak for all five PRC1 proteins was flanked by weaker peaks where only some of the proteins were scored by MACS (for example, *GATA6*, *UNCX* and *NKX2-3* in Figure 2). We interpret these ‘foothill’ peaks as representing weaker or less frequent contacts at the fringes of the main body rather than evidence for specific binding by subsets of PRC1 proteins.

### Validation of PRC1 complex binding at representative loci

To support our interpretation of the ChIP-seq data, we selected representative examples of target loci and performed conventional ChIP followed by quantitative PCR analyses with a series of primers that not only crossed each peak but allowed discrimination between regions of high and low/no enrichment (Additional file 2: Figure S2). The PCR-based assays confirmed the results of the ChIP-seq but additionally showed that, where tested, all of the PRC1 proteins for which we have suitable antibodies had virtually identical binding profiles. These findings are hard to reconcile with the idea that different permutations of PRC1 bind selectivity at different target genes.

### Multiple variants of PRC1 associate with the same DNA

**Figure 3:**
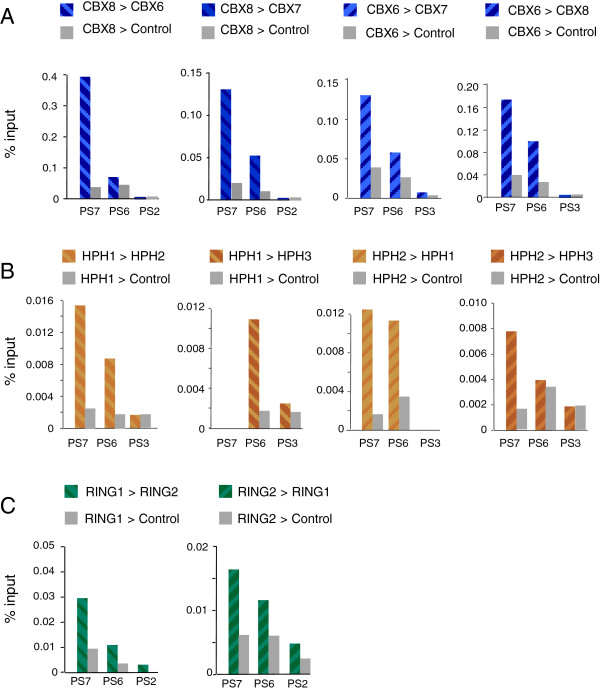
**Sequential ChIP of PRC1 orthologs at the *CDKN2A *locus. (A)** Sequential ChIP was performed with antibodies against CBX8 followed by CBX6 and CBX7, or CBX6 followed by CBX7 and CBX8 as indicated. An irrelevant antibody was used as a control (shown in grey). The recovered chromatin was subjected to quantitative PCR with primers that detect specific PRC1 enrichment at *CDKN2A* (as described in Figure 1). **(B and C)** Equivalent analyses with antibodies against HPH1, HPH2 and HPH3 (B) or RING1 and RING2 (C). ChIP, chromatin immunoprecipitation.

As the ChIP data implied that multiple variants of PRC1 were occupying very similar positions in the genome, it was important to know whether they were simultaneously associated with the same chromatin. To address this question, we performed sequential ChIP with antibodies against different members of the Pc, Ph and Sce families. For example, chromatin precipitated with an antibody against CBX6 was eluted from the Protein-A beads and re-precipitated with antibodies against CBX7, CBX8 or an irrelevant IgG control. The recovered chromatin was then interrogated with PCR primers that demonstrate localized enrichment at *CDKN2A*, and at other loci (Figure 3 and data not shown). Although the analyses were not exhaustive and do not indicate the proportion of each protein that is sequentially precipitated, the data infer that the three CBX proteins can be associated with the same DNA, and that this holds true for the three HPH proteins and both RING proteins. Assuming that the canonical PRC1 complex contains a single representative from each of the component families, and that they can freely assort, the data imply that multiple permutations of PRC1 congregate at common sites throughout the genome.

### Location of PRC1 binding sites relative to potential target genes

Irrespective of the number of PRC1 proteins detected at any one site, an obvious point of interest was the relation between the binding profiles and potential target genes. We compiled a list of candidate loci at which ChIP-seq detected at least one CBX, one RING protein and H3K27me3, using the position of the closest transcription start site (TSS) as the initial criterion. However, a striking feature of the data was that many of the ChIP-seq peaks had a complicated architecture that would be inconsistent with a normal distribution around a single binding site. It was also common to find multiple discrete peaks at the same locus and there was no simple relation between the number of peaks identified by MACS and the number of potential target genes. The bioinformatically compiled lists were therefore edited manually to try to resolve ambiguities (Additional file 3: Table S1). For example, where peaks overlapped adjacent coding and non-coding loci, preference was given to the annotated coding gene.

**Figure 4:**
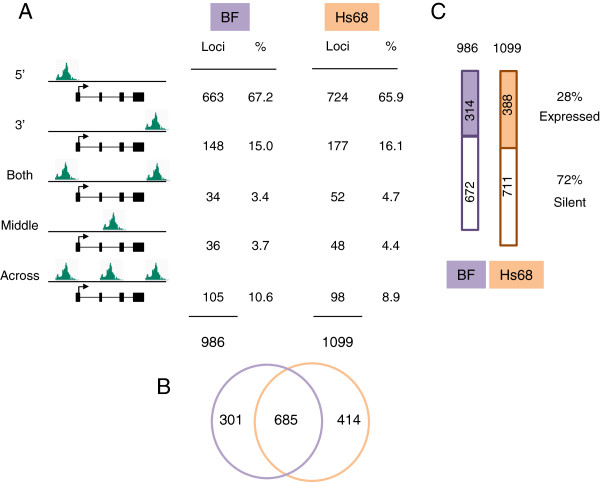
**Location of PRC1 complexes relative to annotated loci. (A)** Diagrammatic representation of the location of ChIP-sequencing peaks relative to a typical gene and the proportions of the peaks that fall into each category in the BF and Hs68 strains of HFs. **(B)** Venn diagram showing the number of potential target genes in the two cell types and the overlap between the datasets. **(C)** Proportions of PRC1 target genes that are actively transcribed in BF and Hs68 cells. ChIP, chromatin immunoprecipitation; HF, human fibroblast.

Most published descriptions of PRC1 and H3K27me3 profiles infer that they are localized in relatively discrete regions within a few kilobase pairs from the TSS. Although this was a common scenario in our target gene list (summarized in Figure 4), the distance from the TSS was very variable (up to 620 kb) and in many cases exceeded the +/- 10 kb range that is typically used in this type of analysis. Importantly, at a substantial number of loci the PRC1 peaks were much closer to a transcription end site (TES) than a TSS. For example, the simple peak just downstream of the *MEIS1* gene is approximately 150 kb from the nearest TSS, which is at the 5' end of *MEIS1* and marked by H3K4me3 (Figure 2B and Additional file 2: Figure S2C). There is no other annotated locus for 500 kb in either direction. We estimated that around 15% to 16% of the potential target loci had peaks that were most closely associated with the 3' end of the transcription unit (Figure 4) but acknowledge that for short genes, this classification is debatable.

The third group of potential targets, representing about 18% of the total, had PRC1 peaks associated with both ends of the transcription unit. However, within this category, there were a number of distinct scenarios (Figure 4). Some loci were flanked by relatively discrete peaks that did not spread across the body of the gene (for example, *UNCX* in Figure 2C), others had internal peaks at some distance from the TSS or TES, but a more common pattern was where peaks were located upstream, downstream and within the transcription unit (for example, *ZIC1* and *ZIC4* in Figure 2D). Where such complicated profiles occurred at closely linked genes, it was not possible to assign the peaks to specific loci.

In conclusion, our data suggest that there is an inherent complexity in the shape of the PRC1 peaks and their location relative to potential target genes. These intricacies in the ChIP-seq profiles have been largely ignored or masked by bioinformatics-based meta-analyses.

### Comparison of the PRC1 binding profiles in different fibroblasts

Despite some residual ambiguities, the peak classification scheme yielded very similar conclusions regarding the numbers of putative target genes in the two strains of HF and the proportions in each category (Figure 4A). A total of 986 loci were scored as candidate PRC1 targets in BF cells compared with 1,099 in Hs68 cells (Additional file 3: Table S1). Importantly, although there were 685 loci in common between the gene lists, 301 were unique to BF cells and 414 were unique to Hs68 cells (Figure 4B).

At the shared loci, the binding patterns were very similar in the two HF strains. This was particularly striking at loci where the peaks had a complicated architecture. Indeed, many of the peaks appeared to resolve into evenly spaced arrays, suggesting some degree of periodicity (for example, *TLX2* and *LBX1* in Figure 2F, G). As the same periodicity was observed with antibodies against all five PRC1 proteins, with independent preparations of chromatin, and in both strains of HF, it seems unlikely to be a technical artefact and warrants further investigation.

**Figure 5:**
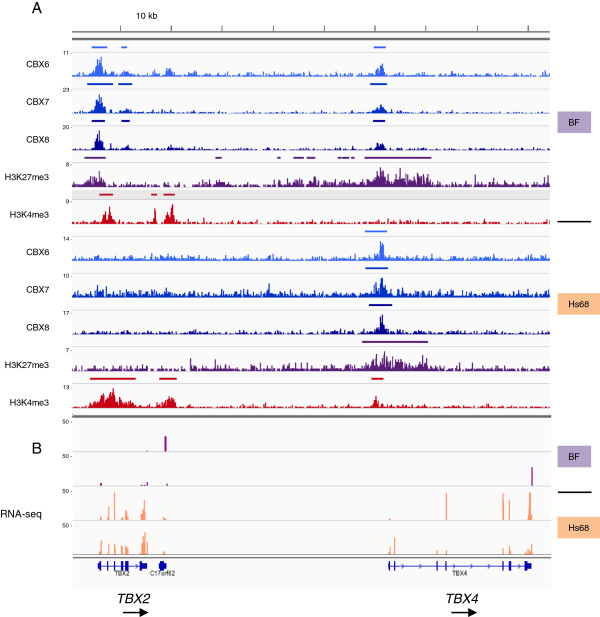
**Correlation of ChIP-sequencing and RNA-sequencing data at the *TBX2 *and *TBX4 *loci. (A)** Profiles of DNA sequence tag densities across the *TBX2* and *TBX4* loci following ChIP-sequencing with the indicated antibodies for BF and Hs68 cells. **(B)** Examples of duplicate RNA-sequencing analyses across the *TBX2* and *TBX4* loci for BF and Hs68 cells. kb, kilobase; RNA-seq, RNA-sequencing.

Where the PRC1 profiles appeared to be strain specific, the differences were generally unambiguous. For example, *TBX2* is a PRC1 target in BF but not Hs68 cells, whereas the adjacent *TBX4* gene is occupied in both (Figure 5 and Additional file 4: Figure S3). Similarly, *NRN1* and *RUNX3* are targets in Hs68 but not BF cells, whereas *MEIS1* shows the reciprocal pattern of occupancy (Additional file 2: Figure S2 and Additional file 4: Figure S3). Among the most striking examples of strain-specific profiles are those for the four HOX gene clusters (Additional file 5: Figure S4). For example, whereas the PRC1 proteins occupy the posterior but not the anterior regions of the *HOX* gene clusters in BF cells, the pattern in Hs68 cells is more complicated, with interspersed binding and non-binding domains across the entire locus.

### Relating PRC1 occupancy to gene expression

**Figure 6:**
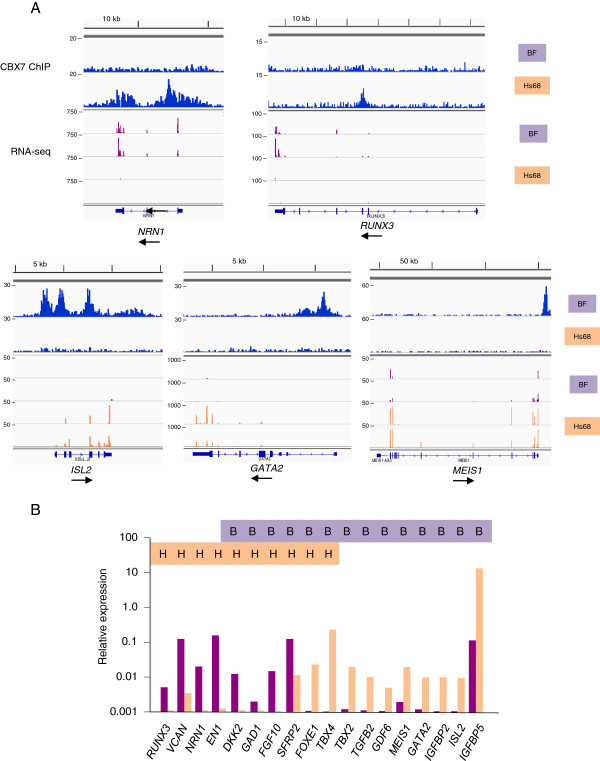
**Correlation of ChIP-sequencing and RNA-sequencing in BF and Hs68 cells. (A)** Comparison of CBX7 binding profiles (top tracks) and RNA-sequencing tag densities at the indicated loci in BF and Hs68 cells. For comparative purposes, the sequence tag densities are plotted on equivalent scales. **(B)** Reverse transcription and quantitative PCR validation of the RNA-sequencing data at the indicated loci. Relative expression levels in BF cells (purple) and Hs68 (tan) cells were calculated relative to RPS17 as an internal standard and plotted on a logarithmic scale. The color-coded B and H symbols above the graph indicate whether the locus is occupied by PRC1 complexes in BF (purple) or Hs68 (tan) cells. Note that *DKK2*, *GAD1*, *FGF10*, *SFRP2*, *FOXE1* and *TBX4* are PRC1 targets in both cell backgrounds. ChIP, chromatin immunoprecipitation; kb, kilobase; RNA-seq, RNA-sequencing.

Although most of the PRC1-occupied loci were devoid of the H3K4me3 mark, consistent with transcriptional repression, a substantial proportion (38%) had an H3K4me3 peak associated with the TSS (for example, *TBX2* and *TBX4* in Figure 5). To explore the relation between PRC1 occupancy and gene expression more directly, we used strand-specific RNA-sequencing (RNA-seq) to profile the transcriptome in BF and Hs68 cells and cross-referenced the resulting datasets with the list of potential target genes compiled from the ChIP-seq. The analyses were conducted on two independently prepared RNA samples from each strain of HF, and an arbitrary threshold of ten read counts in duplicate samples was taken as evidence of active transcription (Additional file 3: Table S1). On this basis, 28% of the PRC1 target genes in each cell type were deemed to be transcriptionally active, whereas 72% were considered silent (Figure 4C). This compares with a 47% to 53% ratio for all expressed and non-expressed genes. At representative loci selected for validation, there was excellent agreement between the results obtained by quantitative reverse transcription PCR (qRT-PCR) and the RNA-seq data. For example, *NRN1* and *RUNX3* were expressed in BF but not Hs68 cells, *ISL2* and *GATA2* were expressed in Hs68 but not BF, whereas *MEIS1* was expressed in both, albeit at different levels (Figure 6). An obvious implication is that the presence of PRC1 at a locus does not necessarily equate with repression, although formal proof would require analyses at the single cell level.

### The genomic landscape of Polycomb binding is maintained at senescence

**Figure 7:**
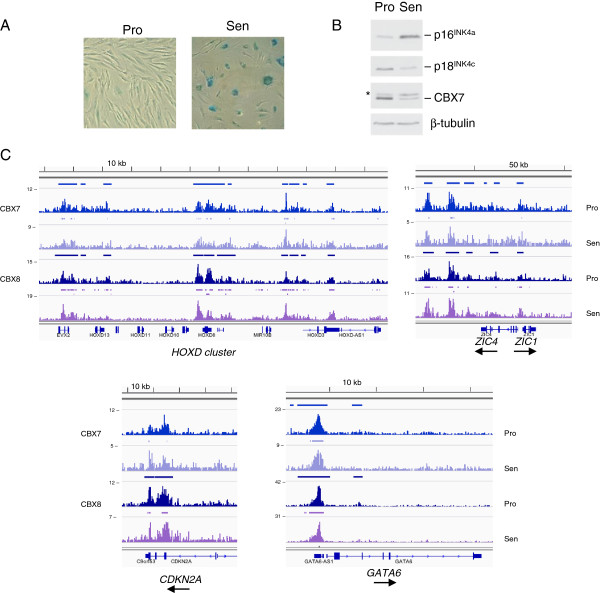
**Genomic landscape of Polycomb orthologs is preserved at senescence. (A)** Staining for senescence-associated β-galactosidase activity in proliferating (Pro) and senescent (Sen) Hs68 cultures. **(B)** Immunoblotting for p16^INK4a^, p18^INK4c^, CBX7 and β-tubulin in proliferating and senescent cells. The asterisk identifies a non-specific band detected with the CBX7 antibody. **(C)** Examples of ChIP-sequencing data obtained for proliferating or senescent Hs68 cells with antibodies against CBX7 and CBX8. Note that the sequence tag densities are not plotted on equivalent scales. kb, kilobase; Pro, proliferating; Sen, senescent.

One of the motivations for profiling Pc orthologs in HFs was to understand the role of PRC1 complexes in the regulation of *CDKN2A* and senescence. We therefore conducted ChIP-seq for CBX7 and CBX8 in Hs68 cells that had been to grown to replicative senescence, as judged by a failure to double after 6 to 8 weeks in culture. As shown in Figure 7A, staining for senescence-associated β-galactosidase (SA-βgal) activity indicated that the cell populations were uniformly senescent and the anticipated accumulation of p16^INK4a^ and decline of p18^INK4c^ levels [30] were confirmed by immunoblotting (Figure 7B). Remarkably, the CBX binding landscapes in the senescent cells were qualitatively indistinguishable from those in proliferating Hs68 cells, in terms of the architecture and locations of the peaks (Figure 7C). However, there was an overall reduction in the density of sequence reads. This was particularly notable for CBX7, potentially reflecting the decline in the total levels of CBX7 in senescent cells (Figure 7B), as previously reported [31]. Thus, although there was no evidence for dramatically altered binding profiles, such as those observed between the two strains of HFs, there could be relatively subtle changes in the amounts of the different Pc orthologs associated with specific loci. Such changes are presumably enough to cause upregulation of p16^INK4a^.

## Discussion

It has been widely assumed that the different variants of PRC1 encoded by mammalian cells are likely to repress different sets of target genes or to substitute for one another in different contexts. Here we suggest a different scenario based on evidence that PRC1 complexes act collectively rather than individually and can be associated with transcriptionally active genes. It is not yet known how many of the potential permutations of the canonical PRC1 complex can be formed in mammalian cells as the existing biochemical data do not provide a strong case for preferential associations between particular subunits. Allowing for free assortment, HFs have the potential to express multiple variants of PRC1 as judged by detection of transcripts for 15 of the 16 core components. We might therefore have expected to see CBX6, 7 and 8 peaks in discrete locations in the ChIP-seq data, in line with recent suggestions [15,24], but instead found that they are largely co-localized. Although there were sites where subsets of the CBX proteins were detected, the signals were generally weaker compared to the loci at which all three CBX proteins were detectable. Likewise, we might have expected to see selective binding of RING1 and RING2, particularly as they can participate in alternative complexes with RYBP and YAF2 but, as others have shown [15-17], they mostly occupied the same positions as the CBX proteins.

As our findings appeared to be odds with recent reports, we re-examined the published datasets to seek a potential explanation, focusing on studies that compared the binding of different members of the same protein family. For example, an extensive survey of 29 chromatin regulators in the K562 human leukemia cell line included data showing that endogenous CBX2 and CBX8 had remarkably coincident binding profiles [32]. Although not explicitly discussed, the inference is that CBX2 and CBX8 have common targets. In contrast, a separate study that compared the binding of epitope-tagged versions of the Psc family in 293T cells concluded that they bind selectively to different categories of target genes [15]. Despite the emphasis on selectivity, the data clearly indicate that a substantial fraction of the target loci were co-occupied by multiple Psc family members. Along similar lines, it was reported that, in mouse embryoid bodies, Cbx2 and Cbx4 bind at both overlapping and non-overlapping sets of target genes [24]. The evidence for specificity was based largely on differences in the sequence read densities achieved with different antibodies and there was considerable variation in the numbers of peaks identified with each reagent. Of note, the data imply that 90% of the Cbx2 peaks overlap with those of Cbx4.

Despite their different interpretations, these published datasets are consistent with the co-localization of PRC1 complexes at multiple sites in the genome. Although we did not detect CBX2 in HFs, we extended the evidence for co-localization to include 11 PRC1 components that, as judged by ChIP-PCR with multiple primer sets, had virtually identical binding patterns at representative loci. Importantly, our ability to perform sequential ChIP with antibodies specific for different members of the Pc, Ph and Sce families argues against the idea that the complexes are acting redundantly in different cells. Rather, a proportion of these complexes must be simultaneously associated with the same DNA.

While our findings do not exclude an element of selectivity in the association of specific PRC1 complexes with specific loci, the overwhelming impression is that they congregate at common sites. One possibility is that they form multimeric assemblies analogous to the Pc bodies described for *Drosophila*[33-35]. Mammalian Pc bodies might comprise multiple permutations of PRC1 rather than multimers of the same complex. Although the ChIP-seq data do not provide a three-dimensional picture, the fact that multiple complexes are co-localized along the genome implies that they are also co-localized in space. To our knowledge, this possibility has not been considered in the existing literature and most discussions about mammalian Pc bodies have centered on their visualization by fluorescence. In primary HFs, endogenous RING1, BMI1 and CBX4 have been detected as multiple speckles that are evenly distributed throughout the cell nucleus, similar to the situation in *Drosophila*[33,36,37] (data not shown). However, these early studies did not address the co-localization of different orthologs or the numbers of complexes that constitute a Pc body. Our attempts to provide such evidence were hampered by a lack of signal for most of the antibodies.

The situation in HFs is in stark contrast to the evidence from human tumor cell lines where the PRC1 proteins are typically found in a small number of very prominent nuclear bodies associated with pericentromeric heterochromatin [36,38-40]. Although multiple PRC1 orthologs are co-localized in these large aggregates [12,41-43], their functional significance has not been established.

Another striking feature of the ChIP-seq data is that the binding profiles have a complicated topography, often with underlying periodicity, that is reproduced with different antibodies, with different preparations of chromatin, and in different cell backgrounds. The baroque nature of the peaks makes it difficult to generalize about their location relative to candidate target genes. Based simply on proximity to the nearest TSS, the ChIP-seq data suggested that PRC1 complexes are co-localized at approximately 1,000 loci in each strain of HFs. However, the distances can be very variable and the patterns do not conform to the idea that PRC1 proteins bind within a few kilobases either side of the TSS, as suggested by meta-analyses of the profiles in ES cells [15,16,24,44].

Focusing on the candidate target genes, we found that a quarter of them are actively transcribed despite the presence of PRC1 in the adjacent DNA. Such observations are not without precedent but PcG complexes are generally viewed as obligate transcriptional repressors [3]. It is formally possible that PRC1 complexes are present on only one allele allowing transcription to occur from the other but another possibility is that the assemblies of PRC1 complexes that we observe are involved in 3D interactions that influence access to both positive and negative transcription factors.

Although there were many parallels regarding the positioning of PRC1 complexes and their effects on gene expression in the two strains of HF, there were several hundred loci at which the binding profiles were completely distinct. Fibroblasts are often considered as a homogeneous cell type but different isolates have different growth rates and sensitivity to senescence [45,46]. Importantly, HFs from different anatomical sites have characteristic patterns of gene expression that reflect their position relative to the developmental axes [47,48]. In this regard, the BF cells would be considered anterior/trunk/non-dermal whereas Hs68 cells would be posterior/distal/dermal [48]. As stromal fibroblasts are thought to influence tissue architecture, via mesenchymal–epithelial interactions, it is interesting to consider whether the signature patterns of PRC1 occupancy represent an ‘epigenetic ZIP-code’.

Senescent fibroblasts also acquire characteristic patterns of gene expression coupled to a plethora of phenotypic and physical alterations [4,5]. At the chromatin level, senescence is accompanied by the formation of senescence-associated heterochromatin foci (SAHFs) that comprise non-overlapping layers of H3K9me3 and H3K27me3 modified chromatin [49-51]. However, a recent study suggested that SAHF formation represents a spatial repositioning of existing marks rather than changes in the landscape of histone modifications in the genome [51]. Consistent with this report, the CBX binding profiles that we observed in proliferating HFs were preserved at senescence and we did not see selective gains or losses of PRC1 peaks that would be comparable to the striking differences between HF strains. Taken together, our findings suggest that the effects of PRC1 complexes on gene expression are not simply dictated by the presence or absence of PRC1 at the locus.

## Conclusions

Our findings question the prevailing view that different variants of PRC1 act on different sets of target genes and suggest that they act collectively rather than individually. Different types of fibroblast have distinctive PRC1 binding landscapes but the landscapes are preserved at senescence. Collectively, our findings suggest that PRC1 complexes have more subtle effects on gene expression than simply blocking transcription of the genes to which they bind.

## Materials and methods

### Cell cultures and immunoblotting

The procedures for propagation of primary human fibroblasts, staining for senescence-associated β-galactosidase activity and immunoblotting were as described [30,46,52].

### Chromatin immunoprecipitation

ChIP assays were performed as described previously [53]. After sonication to obtain chromatin fragments of between 200 and 1000 bp, solubilized chromatin was diluted to 1 μg/μl and incubated with the appropriate antibody at 4°C overnight. The antibodies are listed in Additional file 6: Table S2 and either species-matched irrelevant antibodies or pre-immune serum was used as the control as appropriate. After reversal of the crosslinks, the immunoprecipitated DNA was quantified by real-time PCR with the primer sets described in Additional file 6: Table S3. For sequential ChIP experiments, the eluted chromatin was divided into equal fractions, diluted tenfold in a dilution buffer (0.1% Na deoxycholate, 1% Triton X-100, 1 mM EDTA (ethylenediaminetetraacetic acid), 50 mM HEPES (4-(2-Hydroxyethyl)piperazine-1-ethanesulfonic acid, N-(2-Hydroxyethyl)piperazine-N′-(2-ethanesulfonic acid) pH 7.9, 150 mM NaCl) and subjected to a further round of ChIP with either the same or a different antibody.

### Chromatin immunoprecipitation sequencing and bioinformatic analyses

To generate sufficient quantities of DNA for sequence analyses, parallel ChIP reactions were performed using approximately 5 μg of antibodies with 500 μg chromatin. The recovered material was pooled and concentrated to a minimum of 0.2 μg/μl. DNA samples were end repaired, poly-A tailed and Illumina single-end adapters were ligated following the standard Illumina protocol with minor adjustments. Agencourt AMPure XP beads at 0.8× ratio were used to size-select out adapter dimers after adapter ligation. The Phusion enzyme in the Illumina kit was replaced by the Kapa HiFi HotStart ready mix. Post PCR, AMPure XP beads were used at a 1:1 ratio to maintain size integrity and to allow use of the Invitrogen SizeSelect E-gel system. We found that running the PCR before the gel improved visualization of the product and isolation of the correct band. Samples were finally purified with QIAquick gel extraction kit (Qiagen Ltd) and quality controlled on the DNA 1000 BioAnalyzer 2100 chip before clustering. Input DNA was used as a control for the ChIP-seq analysis. Detailed protocols for library preparation and genome-wide sequencing are available online [54-59].

The alignments were performed using Novoalign (version 2.07.14) [60] allowing for a single mismatch per read. Duplicates reads were removed using the Picard MarkDuplicates program (picard-tools package version 1.48) [61] and peak calling was performed using MACS (version 1.4.0rc2) [29]. The raw and processed data have been deposited under GEO accession number [GEO:GSE40740].

### RNA-sequencing, quantitative reverse transcription and PCR

Total RNA was extracted using the High Pure RNA extraction Kit (Roche). RNA samples were quality controlled (QC) using the 6000 Nano RNA Chip on a BioAnalyzer 2100 (Agilent) and subjected to poly-A selection using Sera-Mag oligo dT beads (Thermo Fisher Scientific Inc). Libraries were prepared using the Directional mRNA-Seq Library Prep. v1.0. Pre-Release Protocol from Illumina with minor adjustments [62]. The Phusion enzyme was replaced by the Kapa HiFi HotStart ready mix which reduced the overall volume of the PCR and the ratio for the Agencourt AMPure XP beads was adjusted accordingly. The standard PCR cycling was also changed to match the concentration of the total RNA from the initial QC. After passing the final QC, the libraries were subjected to cluster formation and then 72-bp single-end sequencing on a GAIIx analyzer [62].

Sequenced reads were aligned using RSEM (version 1.2.4) [63]. Each lane of the GAIIx produced approximately 9 to 20 million unique reads that mapped to RefSeq genes archived in the Illumina iGenomes resource [64].

For qRT-PCR validation, cDNAs were generated from 0.5 to 1 μg of RNA using MultiScribe reverse transcriptase and random hexamer primers (Applied Biosystems). Of the cDNA, 1/50 was used as a template for quantitative PCR with POWER SybrGreen (Applied Biosystems). RPS17 was used as an internal standard. Relative RNA levels were presented either as normalized to RPS17, or as fold-difference between the normalized values. Primer sequences can be found in Additional file 6: Table S4.

## Abbreviations

bp: base pair; ChIP: chromatin immunoprecipitation; ChIP-seq: ChIP-sequencing; ES cell: embryonic stem cell; HF: human fibroblast; kb: kilobase; Pc: Polycomb; PcG: Polycomb group; PCR: polymerase chain reaction; Ph: Polyhomeotic; Psc: Posterior sex combs; QC: quality controlled; qRT-PCR: reverse transcription and quantitative PCR; RNA-seq: RNA-sequencing; SA-βgal: senescence-associated β-galactosidase; SAHF: senescence-associated heterochromatin focus; Sce: Sex combs extra; TES: transcription end site; TSS: transcription start site.

## Competing interests

The authors declare that they have no competing interests.

## Authors’ contributions

HPe, EA, HC and JS performed the ChIP-seq and re-ChIP analyses. RP contributed to the ChIP validation. SB validated the RNA-seq data. MRN, LdeB and TR evaluated the antibodies for specificity and applicability to ChIP. HPa and AS performed the bioinformatic analyses. NM conducted the DNA sequencing. GP conceived the study and wrote the manuscript. All authors read and approved the final manuscript.

## Supplementary Material

Additional file 1: Figure S1 Evaluation of antibody specificity. **(A)** Flag-tagged versions of human CBX2, CBX4, CBX6, CBX7 and CBX8 were expressed in 293T cells by transient transfection of pcDNA6-based vectors. Cell lysates were precipitated with an anti-Flag antibody, ractionated by SDS-PAGE and immunoblotted with the indicated antibodies. Although multiple products were detected, presumably reflecting premature termination or post-translational cleavage, the CBX6 antibodies only detected Flag-CBX6, the CBX7 antibodies only detected Flag-CBX7 and the CBX8 antibodies only detected Flag-CBX8. **(B)** Equivalent analyses with cells expressing Flag-tagged versions of HPH1, HPH2 and HPH3. **(C)** Similar evaluation of antibodies against RING1 and RING2. **(D)** Lysate from cells expressing Flag-tagged CBX4 (left) or CBX6 (right) were immunoprecipitated with antibodies against CBX4, 6, 7 and 8 as indicated. Following SDS-PAGE, the Flag-tagged proteins were identified by immunoblotting. Ig refers to the position of the immunoglobulin heavy chain. Click here for file

Additional file 2: Figure S2 ChIP-PCR analyses of multiple PRC1 proteins at representative loci. Each dataset includes a screenshot of the CBX7 binding profile across the locus (top), with a diagram showing the position of the PCR primer sets relative to the organization of the suspected target gene. The primer sequences are described in Additional file 6: Table S3. The panels show the enrichment observed with the indicated antibody at each primer set as a percentage of input. Grey bars show values for a control IgG antibody. **(A)** GATA6 in BF cells, **(B)** CCND2 in BF cells, **(C)** MEIS1 in BF cells and **(D)** NRN1 in Hs68 cells. Click here for file

Additional file 3: Table S1 List of candidate PRC1 target loci in the BF and Hs68 strains of HF. Alphabetic list of loci associated with PRC1 ChIP-seq peaks in HFs, showing the number of PRC1 proteins identified at the locus for the BF and Hs68 strains. TRUE and FALSE indicate whether the locus is transcriptionally active in BF and Hs68, as judged by RNA-sequencing. Click here for file

Additional file 4: Figure S3 ChIP-PCR showing differential binding of PRC1 proteins in BF and Hs68 cells. Each dataset includes a screenshot of the CBX7 binding profile across the locus (top), with a diagram showing the position of the PCR primer sets relative to the organization of the suspected target gene. The primer sequences are described in Additional file 6: Table S3. The panels show the enrichment observed with the indicated antibody at each primer set as a percentage of input. Grey bars show values for a control IgG antibody. **(A)** TBX2, **(B)** TBX4 and **(C)** RUNX3. Click here for file

Additional file 5: Figure S4 ChIP-seq and RNA-sequencing profiles of the HOX clusters in BF and Hs68 cells. Upper tracks in each figure show the profiles of DNA sequence tag densities following ChIP-seq with antibodies against CBX6, CBX7, CBX8, H3K27me3 and H3K4me3 in the BF and Hs68 strains of HDF as indicated. The lower tracks show duplicate RNA-sequencing data for the corresponding genomic regions in either BF or Hs68 cells. The tag densities were normalized to the same maximum (numbers on left). Click here for file

Additional file 6: Table S2 List of antibodies used for ChIP. **Table S3.** List of oligonucleotide primers for PCR analysis of immunoprecipitated chromatin. **Table S4.** List of oligonucleotide primers used to assess RNA levels by reverse transcription and quantitative PCR. Click here for file

## References

[B1] Muller J Verrijzer P Biochemical mechanisms of gene regulation by polycomb group protein complexes Curr Opin Genet Dev 2009 19 150 158 19345089 10.1016/j.gde.2009.03.001

[B2] Morey L Helin K Polycomb group protein-mediated repression of transcription Trends Biochem Sci 2010 35 323 332 20346678 10.1016/j.tibs.2010.02.009

[B3] Simon JA Kingston RE Occupying chromatin: polycomb mechanisms for getting to genomic targets, stopping transcriptional traffic, and staying put Mol Cell 2013 49 808 824 23473600 10.1016/j.molcel.2013.02.013 PMC3628831

[B4] Campisi J d’Adda di Fagagna F Cellular senescence: when bad things happen to good cells Nat Rev Mol Cell Biol 2007 8 729 740 17667954 10.1038/nrm2233

[B5] Collado M Blasco MA Serrano M Cellular senescence in cancer and aging Cell 2007 130 223 233 17662938 10.1016/j.cell.2007.07.003

[B6] Jacobs JJ Kieboom K Marino S DePinho RA van Lohuizen M The oncogene and Polycomb-group gene *bmi-1* regulates cell proliferation and senescence through the *ink4a* locus Nature 1999 397 164 168 9923679 10.1038/16476

[B7] Maertens GN El Messaoudi-Aubert S Racek T Stock JK Nicholls J Rodriguez-Niedenfuhr M Gil J Peters G Several distinct Polycomb complexes regulate and co-localize on the *INK4a* tumor suppressor locus PLoS One 2009 4 e6380 19636380 10.1371/journal.pone.0006380 PMC2713427

[B8] Whitcomb SJ Basu A Allis CD Bernstein E Polycomb group proteins: an evolutionary perspective Trends Genet 2007 23 494 502 17825942 10.1016/j.tig.2007.08.006

[B9] Shao Z Raible F Mollaaghababa R Guyon JR Wu CT Bender W Kingston RE Stabilization of chromatin structure by PRC1, a Polycomb complex Cell 1999 98 37 46 10412979 10.1016/S0092-8674(00)80604-2

[B10] Saurin AJ Shao Z Erdjument-Bromage H Tempst P Kingston RE A *Drosophila* Polycomb group complex includes Zeste and dTAFII proteins Nature 2001 412 655 660 11493925 10.1038/35088096

[B11] Simon JA Kingston RE Mechanisms of polycomb gene silencing: knowns and unknowns Nat Rev Mol Cell Biol 2009 10 697 708 19738629 10.1038/nrm2763

[B12] Dietrich N Bracken AP Trinh E Schjerling CK Koseki H Rappsilber J Helin K Hansen KH Bypass of senescence by the polycomb group protein CBX8 through direct binding to the INK4A-ARF locus EMBO J 2007 26 1637 1648 17332741 10.1038/sj.emboj.7601632 PMC1829390

[B13] Sanchez C Sanchez I Demmers JA Rodriguez P Strouboulis J Vidal M Proteomics analysis of Ring1B/Rnf2 interactors identifies a novel complex with the Fbxl10/Jhdm1B histone demethylase and the Bcl6 interacting corepressor Mol Cell Proteomics 2007 6 820 834 17296600 10.1074/mcp.M600275-MCP200

[B14] Vandamme J Volkel P Rosnoblet C Le Faou P Angrand PO Interaction proteomics analysis of polycomb proteins defines distinct PRC1 complexes in mammalian cells Mol Cell Proteomics 2011 10 M110 002642 21282530 10.1074/mcp.M110.002642 PMC3069339

[B15] Gao Z Zhang J Bonasio R Strino F Sawai A Parisi F Kluger Y Reinberg D PCGF homologs, CBX proteins, and RYBP define functionally distinct PRC1 family complexes Mol Cell 2012 45 344 356 22325352 10.1016/j.molcel.2012.01.002 PMC3293217

[B16] Tavares L Dimitrova E Oxley D Webster J Poot R Demmers J Bezstarosti K Taylor S Ura H Koide H Wutz A Vidal M Elderkin S Brockdorff N RYBP-PRC1 complexes mediate H2A ubiquitylation at Polycomb target sites independently of PRC2 and H3K27me3 Cell 2012 148 664 678 22325148 10.1016/j.cell.2011.12.029 PMC3281992

[B17] Morey L Aloia L Cozzuto L Benitah SA Di Croce L RYBP and Cbx7 define specific biological functions of polycomb complexes in mouse embryonic stem cells Cell Rep 2013 3 60 69 23273917 10.1016/j.celrep.2012.11.026

[B18] Eskeland R Leeb M Grimes GR Kress C Boyle S Sproul D Gilbert N Fan Y Skoultchi AI Wutz A Bickmore WA Ring1B compacts chromatin structure and represses gene expression independent of histone ubiquitination Mol Cell 2010 38 452 464 20471950 10.1016/j.molcel.2010.02.032 PMC3132514

[B19] Gutierrez L Oktaba K Scheuermann JC Gambetta MC Ly-Hartig N Muller J The role of the histone H2A ubiquitinase Sce in Polycomb repression Development 2012 139 117 127 22096074 10.1242/dev.074450 PMC3253035

[B20] Wang R Taylor AB Leal BZ Chadwell LV Ilangovan U Robinson AK Schirf V Hart PJ Lafer EM Demeler B Hinck AP McEwen DG Kim CA Polycomb group targeting through different binding partners of RING1B C-terminal domain Structure 2010 18 966 975 20696397 10.1016/j.str.2010.04.013 PMC4445678

[B21] Bernstein E Duncan EM Masui O Gil J Heard E Allis CD Mouse polycomb proteins bind differentially to methylated histone H3 and RNA and are enriched in facultative heterochromatin Mol Cell Biol 2006 26 2560 2569 16537902 10.1128/MCB.26.7.2560-2569.2006 PMC1430336

[B22] Kagey MH Melhuish TA Wotton D The polycomb protein Pc2 is a SUMO E3 Cell 2003 113 127 137 12679040 10.1016/s0092-8674(03)00159-4

[B23] Kaustov L Ouyang H Amaya M Lemak A Nady N Duan S Wasney GA Li Z Vedadi M Schapira M Min J Arrowsmith CH Recognition and specificity determinants of the human cbx chromodomains J Biol Chem 2011 286 521 529 21047797 10.1074/jbc.M110.191411 PMC3013012

[B24] Morey L Pascual G Cozzuto L Roma G Wutz A Benitah SA Di Croce L Nonoverlapping functions of the polycomb group cbx family of proteins in embryonic stem cells Cell Stem Cell 2012 10 47 62 22226355 10.1016/j.stem.2011.12.006

[B25] O'Loghlen A Munoz-Cabello AM Gaspar-Maia A Wu HA Banito A Kunowska N Racek T Pemberton HN Beolchi P Lavial F Masui O Vermeulen M Carroll T Graumann J Heard E Dillon N Azuara V Snijders AP Peters G Bernstein E Gil J MicroRNA regulation of Cbx7 mediates a switch of Polycomb orthologs during ESC differentiation Cell Stem Cell 2012 10 33 46 22226354 10.1016/j.stem.2011.12.004 PMC3277884

[B26] Gil J Peters G Regulation of the INK4b-ARF-INK4a tumour suppressor locus: all for one or one for all Nat Rev Mol Cell Biol 2006 7 667 677 16921403 10.1038/nrm1987

[B27] Bracken AP Kleine-Kohlbrecher D Dietrich N Pasini D Gargiulo G Beekman C Theilgaard-Monch K Minucci S Porse BT Marine JC Hansen KH Helin K The Polycomb group proteins bind throughout the INK4A-ARF locus and are disassociated in senescent cells Genes Dev 2007 21 525 530 17344414 10.1101/gad.415507 PMC1820894

[B28] Gene Expression Ominbus (GEO) [http://www.ncbi.nlm.nih.gov/geo/query/acc.cgi?acc=GSE40740]

[B29] Zhang Y Liu T Meyer CA Eeckhoute J Johnson DS Bernstein BE Nusbaum C Myers RM Brown M Li W Liu XS Model-based analysis of ChIP-Seq (MACS) Genome Biol 2008 9 R137 18798982 10.1186/gb-2008-9-9-r137 PMC2592715

[B30] Gagrica S Brookes S Anderton E Rowe J Peters G Contrasting behavior of the p18INK4c and p16INK4a tumor suppressors in both replicative and oncogene-induced senescence Cancer Res 2012 72 165 175 22080569 10.1158/0008-5472.CAN-11-2552 PMC4248231

[B31] Gil J Bernard D Martinez D Beach D Polycomb CBX7 has a unifying role in cellular lifespan Nat Cell Biol 2004 6 67 72 14647293 10.1038/ncb1077

[B32] Ram O Goren A Amit I Shoresh N Yosef N Ernst J Kellis M Gymrek M Issner R Coyne M Durham Tm Zhang X Donaghey J Epstein CB Regev A Bernstein BE Combinatorial patterning of chromatin regulators uncovered by genome-wide location analysis in human cells Cell 2011 147 1628 1639 22196736 10.1016/j.cell.2011.09.057 PMC3312319

[B33] Buchenau P Hodgson J Strutt H Arndt-Jovin DJ The distribution of polycomb-group proteins during cell division and development in *Drosophila* embryos: impact on models for silencing J Cell Biol 1998 141 469 481 9548724 10.1083/jcb.141.2.469 PMC2148446

[B34] Messmer S Franke A Paro R Analysis of the functional role of the Polycomb chromo domain in *Drosophila melanogaster* Genes Dev 1992 6 1241 1254 1628830 10.1101/gad.6.7.1241

[B35] Pirrotta V Li HB A view of nuclear Polycomb bodies Curr Opin Genet Dev 2011 22 101 109 22178420 10.1016/j.gde.2011.11.004 PMC3329586

[B36] Saurin AJ Shiels C Williamson J Satijn DP Otte AP Sheer D Freemont PS The human polycomb group complex associates with pericentromeric heterochromatin to form a novel nuclear domain J Cell Biol 1998 142 887 898 9722603 10.1083/jcb.142.4.887 PMC2132874

[B37] Voncken JW Schweizer D Aagaard L Sattler L Jantsch MF van Lohuizen M Chromatin-association of the Polycomb group protein BMI1 is cell cycle-regulated and correlates with its phosphorylation status J Cell Sci 1999 112 4627 4639 10574711 10.1242/jcs.112.24.4627

[B38] Alkema MJ Bronk M Verhoeven E Otte A van’t Veer LJ Berns A van Lohuizen M Identification of Bmi1-interacting proteins as constituents of a multimeric mammalian polycomb complex Genes Dev 1997 11 226 240 9009205 10.1101/gad.11.2.226

[B39] Cmarko D Verschure PJ Otte AP van Driel R Fakan S Polycomb group gene silencing proteins are concentrated in the perichromatin compartment of the mammalian nucleus J Cell Sci 2003 116 335 343 12482919 10.1242/jcs.00225

[B40] Hernandez-Munoz I Taghavi P Kuijl C Neefjes J van Lohuizen M Association of BMI1 with polycomb bodies is dynamic and requires PRC2/EZH2 and the maintenance DNA methyltransferase DNMT1 Mol Cell Biol 2005 25 11047 11058 16314526 10.1128/MCB.25.24.11047-11058.2005 PMC1316945

[B41] Garcia E Marcos-Gutierrez C del Mar Lorente M Moreno JC Vidal M RYBP, a new repressor protein that interacts with components of the mammalian Polycomb complex, and with the transcription factor YY1 EMBO J 1999 18 3404 3418 10369680 10.1093/emboj/18.12.3404 PMC1171420

[B42] Atsuta T Fujimura S Moriya H Vidal M Akasaka T Koseki H Production of monoclonal antibodies against mammalian Ring1B proteins Hybridoma 2001 20 43 46 11289226 10.1089/027245701300060427

[B43] Satijn DP Olson DJ van der Vlag J Hamer KM Lambrechts C Masselink H Gunster MJ Sewalt RG van Driel R Otte AP Interference with the expression of a novel human polycomb protein, hPc2, results in cellular transformation and apoptosis Mol Cell Biol 1997 17 6076 6086 9315667 10.1128/mcb.17.10.6076 PMC232457

[B44] Ku M Koche RP Rheinbay E Mendenhall EM Endoh M Mikkelsen TS Presser A Nusbaum C Xie X Chi AS Adli M Kasif S Ptaszek LM Cowan CA Koseki H Bernstein BE Genomewide analysis of PRC1 and PRC2 occupancy identifies two classes of bivalent domains PLoS Genet 2008 4 e1000242 18974828 10.1371/journal.pgen.1000242 PMC2567431

[B45] Itahana K Zou Y Itahana Y Martinez JL Beausejour C Jacobs JJ Van Lohuizen M Band V Campisi J Dimri GP Control of the replicative life span of human fibroblasts by p16 and the polycomb protein Bmi-1 Mol Cell Biol 2003 23 389 401 12482990 10.1128/MCB.23.1.389-401.2003 PMC140680

[B46] Brookes S Rowe J Gutierrez Del Arroyo A Bond J Peters G Contribution of p16(INK4a) to replicative senescence of human fibroblasts Exp Cell Res 2004 298 549 559 15265701 10.1016/j.yexcr.2004.04.035

[B47] Chang HY Chi JT Dudoit S Bondre C van de Rijn M Botstein D Brown PO Diversity, topographic differentiation, and positional memory in human fibroblasts Proc Natl Acad Sci U S A 2002 99 12877 12882 12297622 10.1073/pnas.162488599 PMC130553

[B48] Rinn JL Bondre C Gladstone HB Brown PO Chang HY Anatomic demarcation by positional variation in fibroblast gene expression programs PLoS Genet 2006 2 e119 16895450 10.1371/journal.pgen.0020119 PMC1523235

[B49] Narita M Nunez S Heard E Narita M Lin AW Hearn SA Spector DL Hannon GJ Lowe SW Rb-mediated heterochromatin formation and silencing of E2F target genes during cellular senescence Cell 2003 113 703 716 12809602 10.1016/s0092-8674(03)00401-x

[B50] Zhang R Chen W Adams PD Molecular dissection of formation of senescence-associated heterochromatin foci Mol Cell Biol 2007 27 2343 2358 17242207 10.1128/MCB.02019-06 PMC1820509

[B51] Chandra T Kirschner K Thuret JY Pope BD Ryba T Newman S Ahmed K Samarajiwa SA Salama R Carroll T Stark R Janky R Narita M Xue L Chicas A Nunez S Janknecht R Hayashi-Takanaka Y Wilson MD Marshall A Odom DT Babu MM Bazett-Jones DP Tavare S Edwards PA Lowe SW Kimura H Gilbert DM Narita M Independence of repressive histone marks and chromatin compaction during senescent heterochromatic layer formation Mol Cell 2012 47 203 214 22795131 10.1016/j.molcel.2012.06.010 PMC3701408

[B52] Barradas M Anderton E Acosta JC Li S Banito A Rodriguez-Niedenfuhr M Maertens G Banck M Zhou MM Walsh MJ Peters G Gil J Histone demethylase JMJD3 contributes to epigenetic control of INK4a/ARF by oncogenic RAS Genes Dev 2009 23 1177 1182 19451218 10.1101/gad.511109 PMC2685533

[B53] Stock JK Giadrossi S Casanova M Brookes E Vidal M Koseki H Brockdorff N Fisher AG Pombo A Ring1-mediated ubiquitination of H2A restrains poised RNA polymerase II at bivalent genes in mouse ES cells Nat Cell Biol 2007 9 1428 1435 18037880 10.1038/ncb1663

[B54] Preparing samples for ChIP sequencing of DNA [http://genome.med.harvard.edu/documents/illumina/11257047_ChIP_Sample_Prep.pdf]

[B55] E-Gel® SizeSelect™ Agarose Gels [http://tools.invitrogen.com/content/sfs/manuals/egel_sizeselect_qrc.pdf]

[B56] Agencourt® AMPure® PCR Purification [http://genome.med.harvard.edu/documents/sequencing/Agencourt_AMPure_Protocol.pdf]

[B57] Illumina TruSeq cluster generation protocol [https://icom.illumina.com/download/summary/m3k7sKMtGEOX1RjuSLQzjQ]

[B58] Illumina TruSeq reagent protocol [https://icom.illumina.com/download/summary/PRv_NCP-DEaVrFfTbSdQnw]

[B59] Illumina GAIIx user protocol [https://icom.illumina.com/download/summary/NhgBUf-w1USCWAimIVWgTA]

[B60] Novocvraft [http://novocraft.com]

[B61] Picard [http://picard.sourceforge.net]

[B62] Directional mRNA-Seq Library Prep. Pre-Release Protocol [http://corelabs.cgrb.oregonstate.edu/sites/default/files/file/hts/Directional-RNA-Seq-Experienced-User-Card%5B1%5D.pdf]

[B63] Li B Dewey CN RSEM: accurate transcript quantification from RNA-seq data with or without a reference genome BMC Bioinformatics 2011 12 323 21816040 10.1186/1471-2105-12-323 PMC3163565

[B64] Illumina iGenomes [http://support.illumina.com/sequencing/sequencing_software/igenome.ilmn]

