# Supplementary material for: Genome-wide co-localization of Polycomb orthologs and their effects on gene expression in human fibroblasts
Source: Genome Biol. 2014 Feb 3;15(2):R23. doi: 10.1186/gb-2014-15-2-r23 (PMC4053772; doi:10.1186/gb-2014-15-2-r23)
Supplement: Additional file 1: Figure S1 — Evaluation of antibody specificity. (A) Flag-tagged versions of human CBX2, CBX4, CBX6, CBX7 and CBX8 were expressed in 293T cells by transient transfection of pcDNA6-based vectors. Cell lysates were precipitated with an anti-Flag antibody, ractionated by SDS-PAGE and immunoblotted with the indicated antibodies. Although multiple products were detected, presumably reflecting premature termination or post-translational cleavage, the CBX6 antibodies only detected Flag-CBX6, the CBX7 antibodies only detected Flag-CBX7 and the CBX8 antibodies only detected Flag-CBX8. (B) Equivalent analyses with cells expressing Flag-tagged versions of HPH1, HPH2 and HPH3. (C) Similar evaluation of antibodies against RING1 and RING2. (D) Lysate from cells expressing Flag-tagged CBX4 (left) or CBX6 (right) were immunoprecipitated with antibodies against CBX4, 6, 7 and 8 as indicated. Following SDS-PAGE, the Flag-tagged proteins were identified by immunoblotting. Ig refers to the position of the immunoglobulin heavy chain. [file gb-2014-15-2-r23-S1.ppt]

## Slide 1
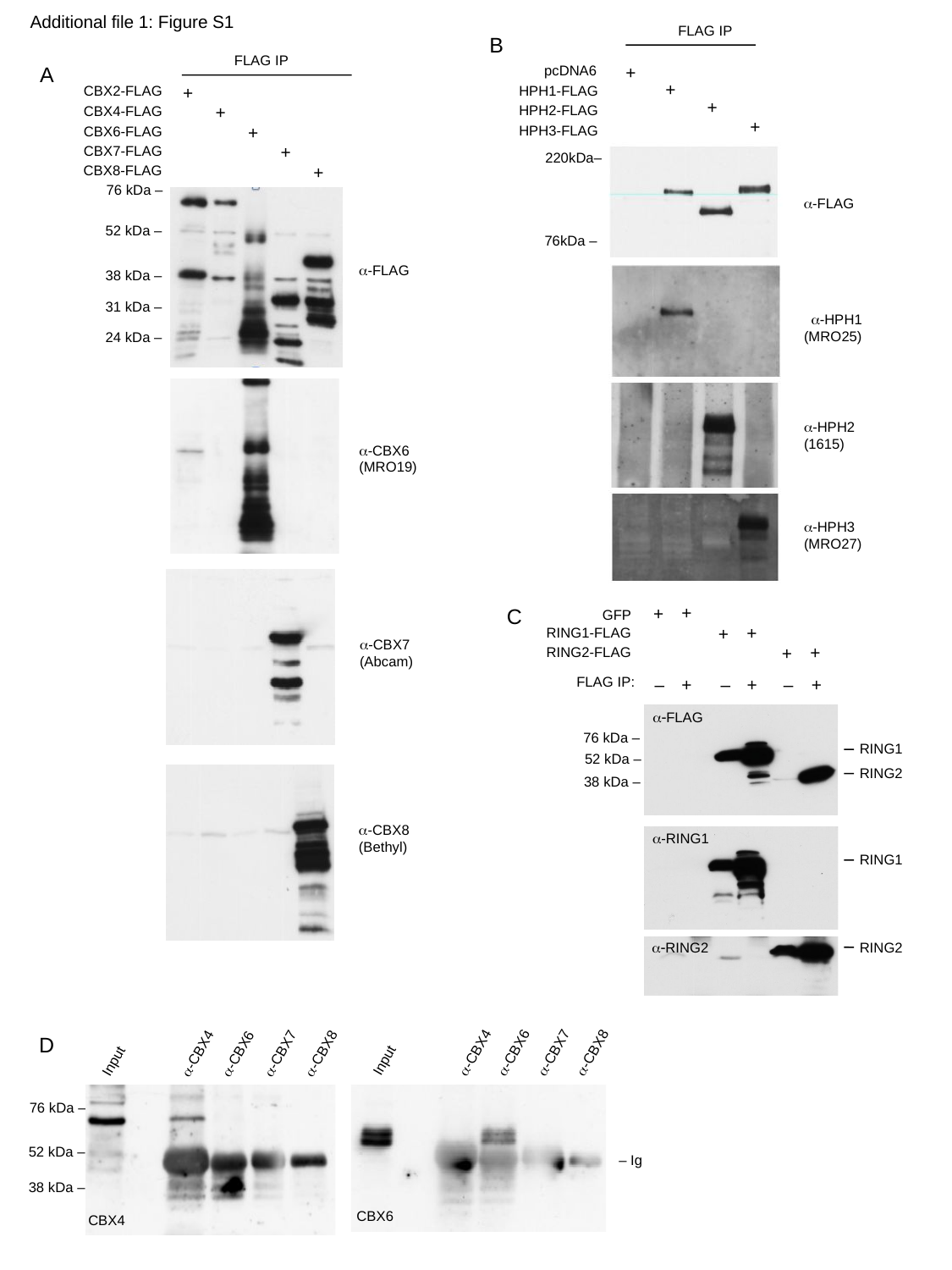

Additional file 1: Figure S1
FLAG IP
B
FLAG IP
A
pcDNA6
+
+
+
HPH1-FLAG
CBX2-FLAG
+
+
HPH2-FLAG
CBX4-FLAG
+
+
HPH3-FLAG
CBX6-FLAG
+
CBX7-FLAG
220kDa–
CBX8-FLAG
+
76 kDa –
α-FLAG
52 kDa –
76kDa –
α-FLAG
38 kDa –
31 kDa –
α-HPH1
(MRO25)
24 kDa –
α-HPH2
(1615)
α-CBX6
(MRO19)
α-HPH3
(MRO27)
+
+
C
GFP
+
+
RING1-FLAG
+
+
RING2-FLAG
FLAG IP:
–
+
–
+
–
+
α−FLAG
76 kDa –
RING1
52 kDa –
RING2
38 kDa –
α-RING1
RING1
RING2
α−RING2
α-CBX7
(Abcam)
α-CBX8
(Bethyl)
α-CBX4
α-CBX6
α-CBX7
α-CBX8
Input
α-CBX4
α-CBX6
α-CBX7
α-CBX8
Input
D
76 kDa –
52 kDa –
– Ig
38 kDa –
CBX6
CBX4
