# Supplementary material for: Genome-wide co-localization of Polycomb orthologs and their effects on gene expression in human fibroblasts
Source: Genome Biol. 2014 Feb 3;15(2):R23. doi: 10.1186/gb-2014-15-2-r23 (PMC4053772; doi:10.1186/gb-2014-15-2-r23)
Supplement: Additional file 2: Figure S2 — ChIP-PCR analyses of multiple PRC1 proteins at representative loci. Each dataset includes a screenshot of the CBX7 binding profile across the locus (top), with a diagram showing the position of the PCR primer sets relative to the organization of the suspected target gene. The primer sequences are described in Additional file 6: Table S3. The panels show the enrichment observed with the indicated antibody at each primer set as a percentage of input. Grey bars show values for a control IgG antibody. (A) GATA6 in BF cells, (B) CCND2 in BF cells, (C) MEIS1 in BF cells and (D) NRN1 in Hs68 cells. [file gb-2014-15-2-r23-S2.pptx]

## Slide 1
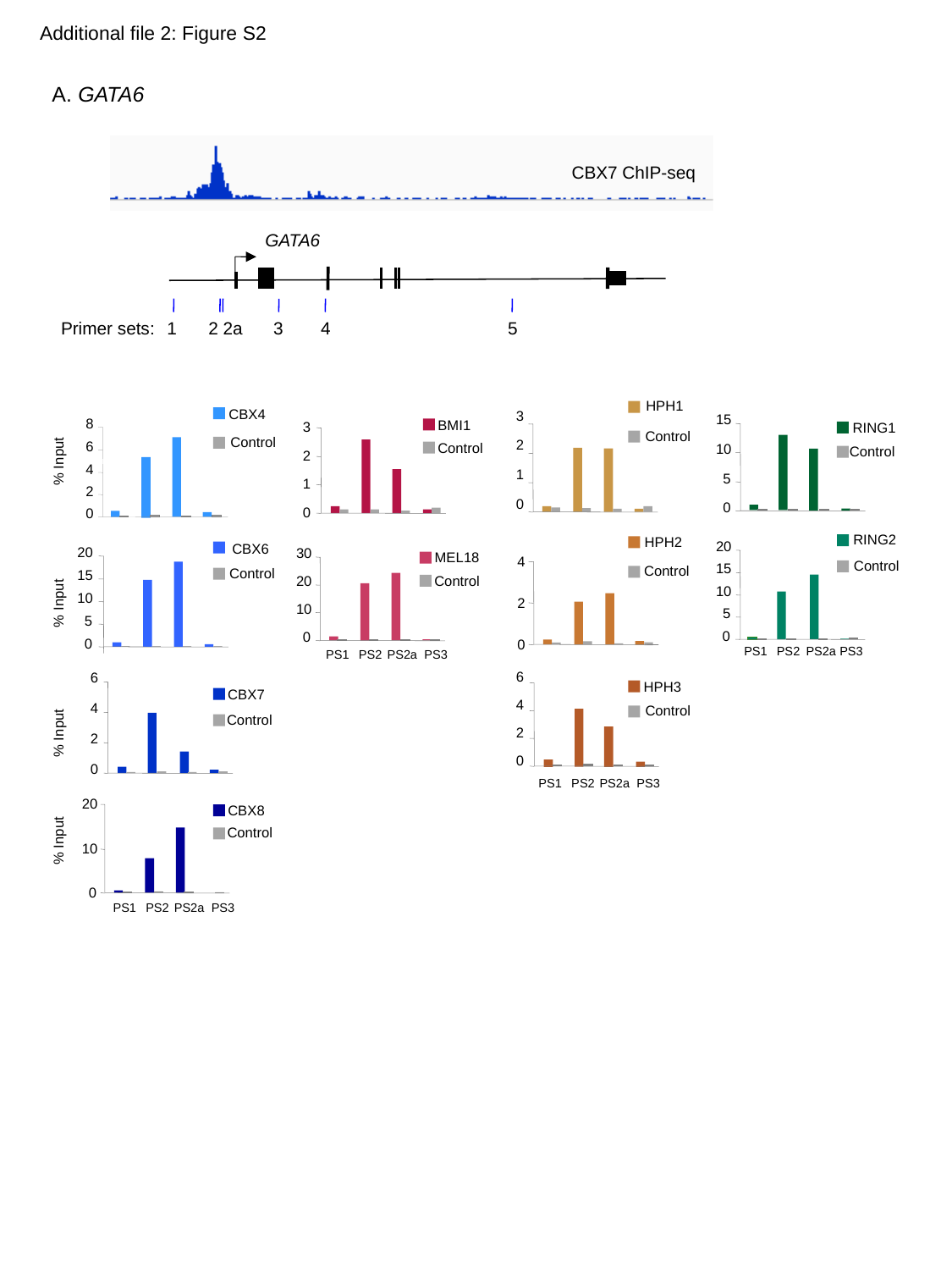

Additional file 2: Figure S2
A. GATA6
CBX7 ChIP-seq
GATA6
Primer sets:
1
2 2a
3
4
5
HPH1
3
Control
2
1
0
CBX4
8
Control
6
4
2
0
15
RING1
10
Control
5
0
BMI1
3
Control
2
1
0
% Input
RING2
20
Control
15
10
5
0
PS1
PS2
PS2a
PS3
HPH2
4
Control
2
0
CBX6
20
Control
15
10
5
0
30
MEL18
20
Control
10
0
PS1
PS2
PS2a
PS3
% Input
6
HPH3
4
Control
2
0
6
CBX7
4
Control
2
0
% Input
PS1
PS2
PS2a
PS3
20
CBX8
Control
% Input
10
0
PS1
PS2
PS2a
PS3

## Slide 2
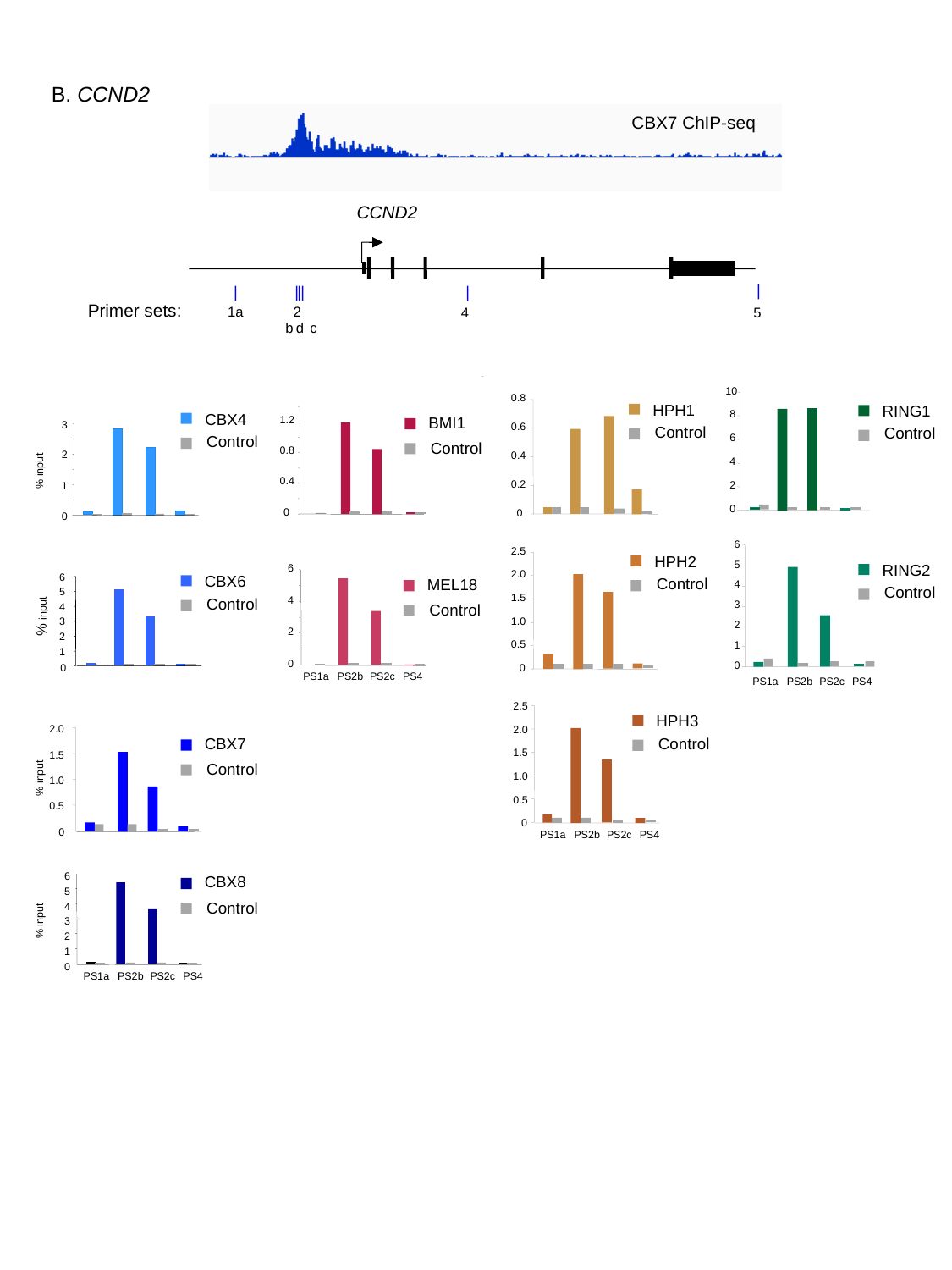

B. CCND2
CBX7 ChIP-seq
CCND2
Primer sets:
2
1a
4
5
b
d
c
10
RING1
8
Control
6
4
2
0
0.8
HPH1
0.6
Control
0.4
0.2
0
BMI1
1.2
Control
0.8
0.4
0
CBX4
3
Control
2
% input
1
0
6
5
RING2
4
Control
3
2
1
0
PS1a
PS2b
PS2c
PS4
2.5
HPH2
2.0
Control
1.5
1.0
0.5
0
6
MEL18
4
Control
2
0
PS1a
PS2b
PS2c
PS4
6
CBX6
5
Control
4
% input
3
2
1
0
2.5
HPH3
2.0
Control
1.5
1.0
0.5
0
PS1a
PS2b
PS2c
PS4
2.0
CBX7
1.5
Control
% input
1.0
0.5
0
6
CBX8
5
Control
4
% input
3
2
1
0
PS1a
PS2b
PS2c
PS4

## Slide 3
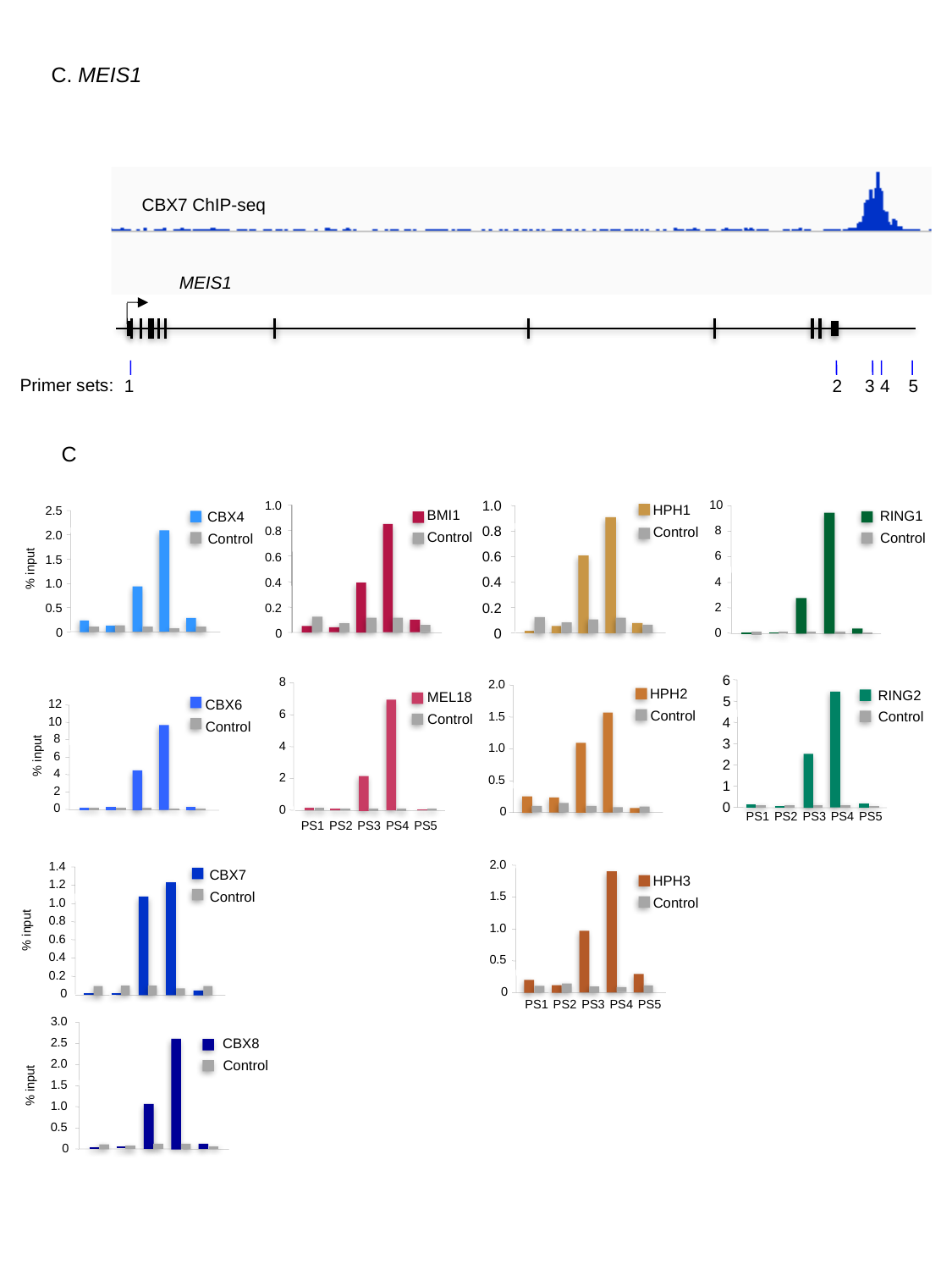

C. MEIS1
CBX7 ChIP-seq
MEIS1
Primer sets:
1
2
3
4
5
C
1.0
HPH1
0.8
Control
0.6
0.4
0.2
0
10
RING1
8
Control
6
4
2
0
1.0
BMI1
0.8
Control
0.6
0.4
0.2
0
2.5
CBX4
2.0
Control
1.5
% input
1.0
0.5
0
6
RING2
5
Control
4
3
2
1
0
PS1
PS2
PS3
PS4
PS5
8
MEL18
6
Control
4
2
0
PS1
PS2
PS3
PS4
PS5
2.0
HPH2
Control
1.5
1.0
0.5
0
CBX6
12
10
Control
8
% input
6
4
2
0
2.0
HPH3
1.5
Control
1.0
0.5
0
PS1
PS2
PS3
PS4
PS5
1.4
CBX7
1.2
Control
1.0
0.8
% input
0.6
0.4
0.2
0
3.0
CBX8
2.5
2.0
Control
1.5
% input
1.0
0.5
0

## Slide 4
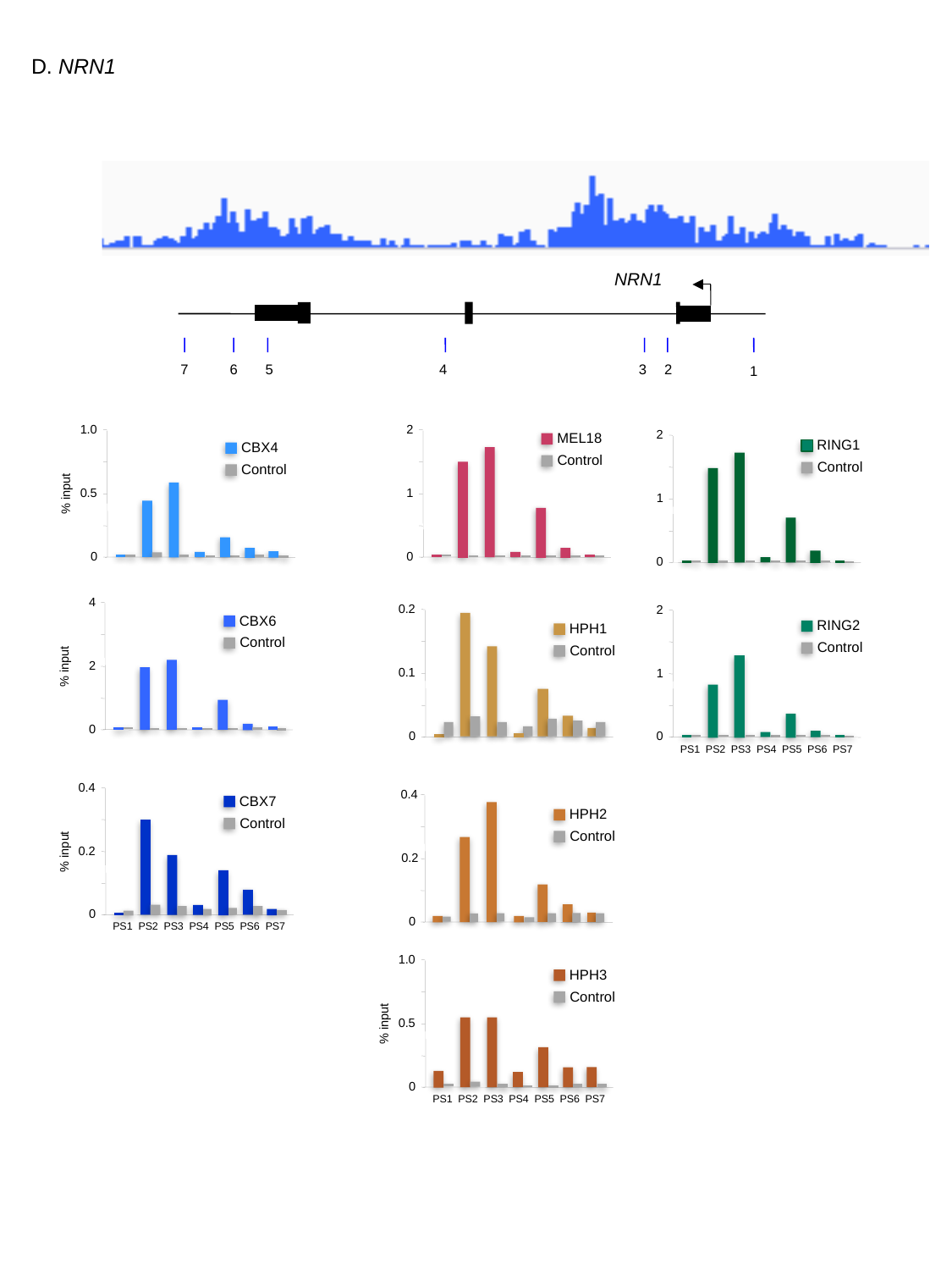

D. NRN1
NRN1
7
6
5
4
3
2
1
1.0
CBX4
Control
0.5
% input
0
2
MEL18
Control
1
0
2
RING1
Control
1
0
4
CBX6
Control
2
% input
0
0.2
HPH1
Control
0.1
0
2
RING2
Control
1
0
PS1
PS2
PS3
PS4
PS5
PS6
PS7
0.4
CBX7
Control
0.2
% input
0
PS1
PS2
PS3
PS4
PS5
PS6
PS7
0.4
HPH2
Control
0.2
0
1.0
HPH3
Control
0.5
% input
0
PS1
PS2
PS3
PS4
PS5
PS6
PS7
