# Supplementary material for: Genome-wide co-localization of Polycomb orthologs and their effects on gene expression in human fibroblasts
Source: Genome Biol. 2014 Feb 3;15(2):R23. doi: 10.1186/gb-2014-15-2-r23 (PMC4053772; doi:10.1186/gb-2014-15-2-r23)
Supplement: Additional file 4: Figure S3 — ChIP-PCR showing differential binding of PRC1 proteins in BF and Hs68 cells. Each dataset includes a screenshot of the CBX7 binding profile across the locus (top), with a diagram showing the position of the PCR primer sets relative to the organization of the suspected target gene. The primer sequences are described in Additional file 6: Table S3. The panels show the enrichment observed with the indicated antibody at each primer set as a percentage of input. Grey bars show values for a control IgG antibody. (A) TBX2, (B) TBX4 and (C) RUNX3. [file gb-2014-15-2-r23-S4.pptx]

## Slide 1
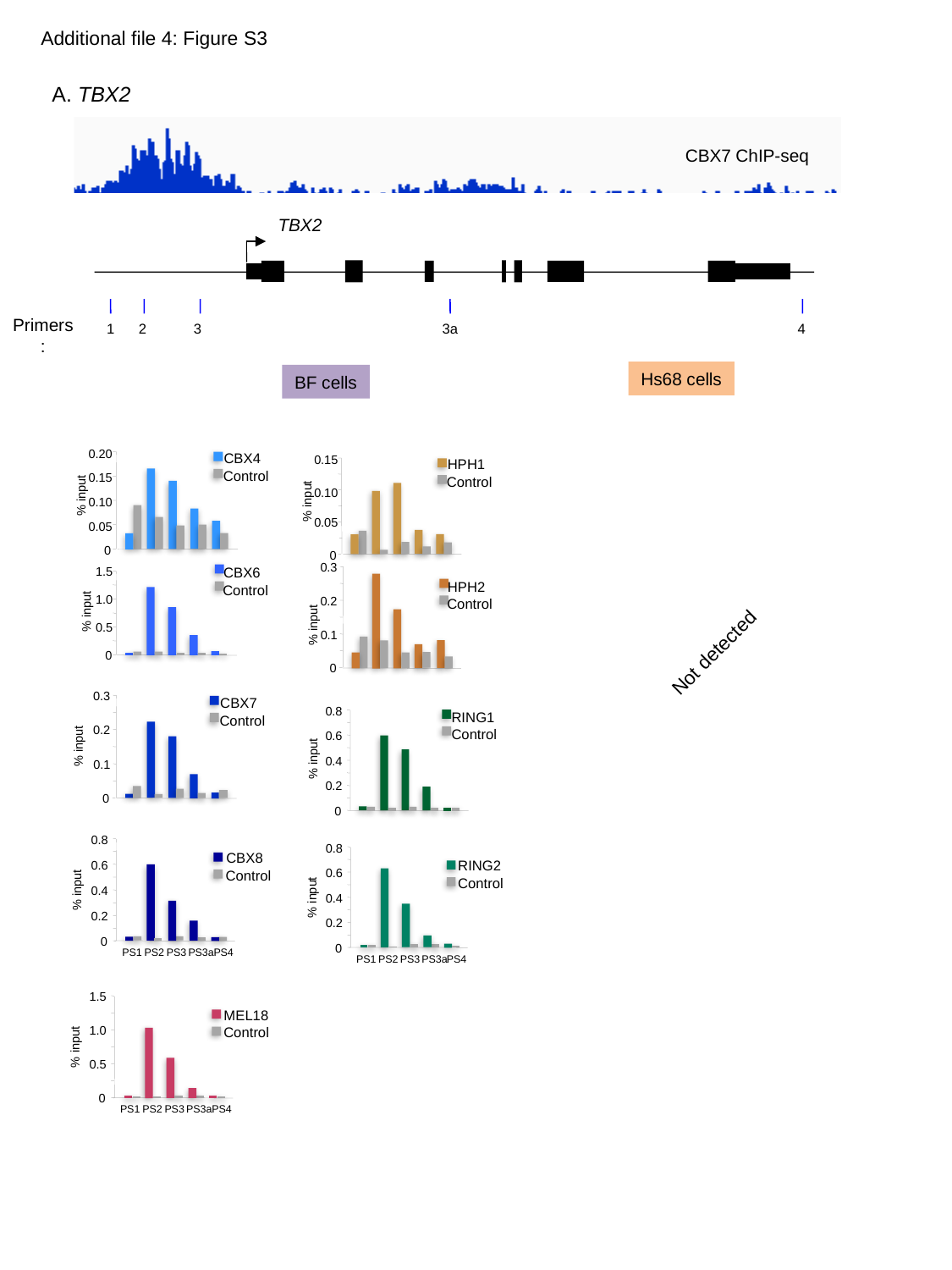

Additional file 4: Figure S3
A. TBX2
CBX7 ChIP-seq
TBX2
Primers:
1
2
3
3a
4
Hs68 cells
BF cells
0.20
CBX4
Control
0.15
% input
0.10
0.05
0
CBX6
1.5
Control
1.0
% input
0.5
0
0.3
CBX7
Control
0.2
% input
0.1
0
0.8
CBX8
0.6
Control
0.4
% input
0.2
0
PS1
PS2
PS3
PS3a
PS4
1.5
MEL18
1.0
Control
% input
0.5
0
PS1
PS2
PS3
PS3a
PS4
0.15
HPH1
Control
0.10
% input
0.05
0
0.3
HPH2
0.2
Control
% input
0.1
0
0.8
RING1
Control
0.6
% input
0.4
0.2
0
0.8
RING2
0.6
Control
0.4
% input
0.2
0
PS1
PS2
PS3
PS3a
PS4
Not detected

## Slide 2
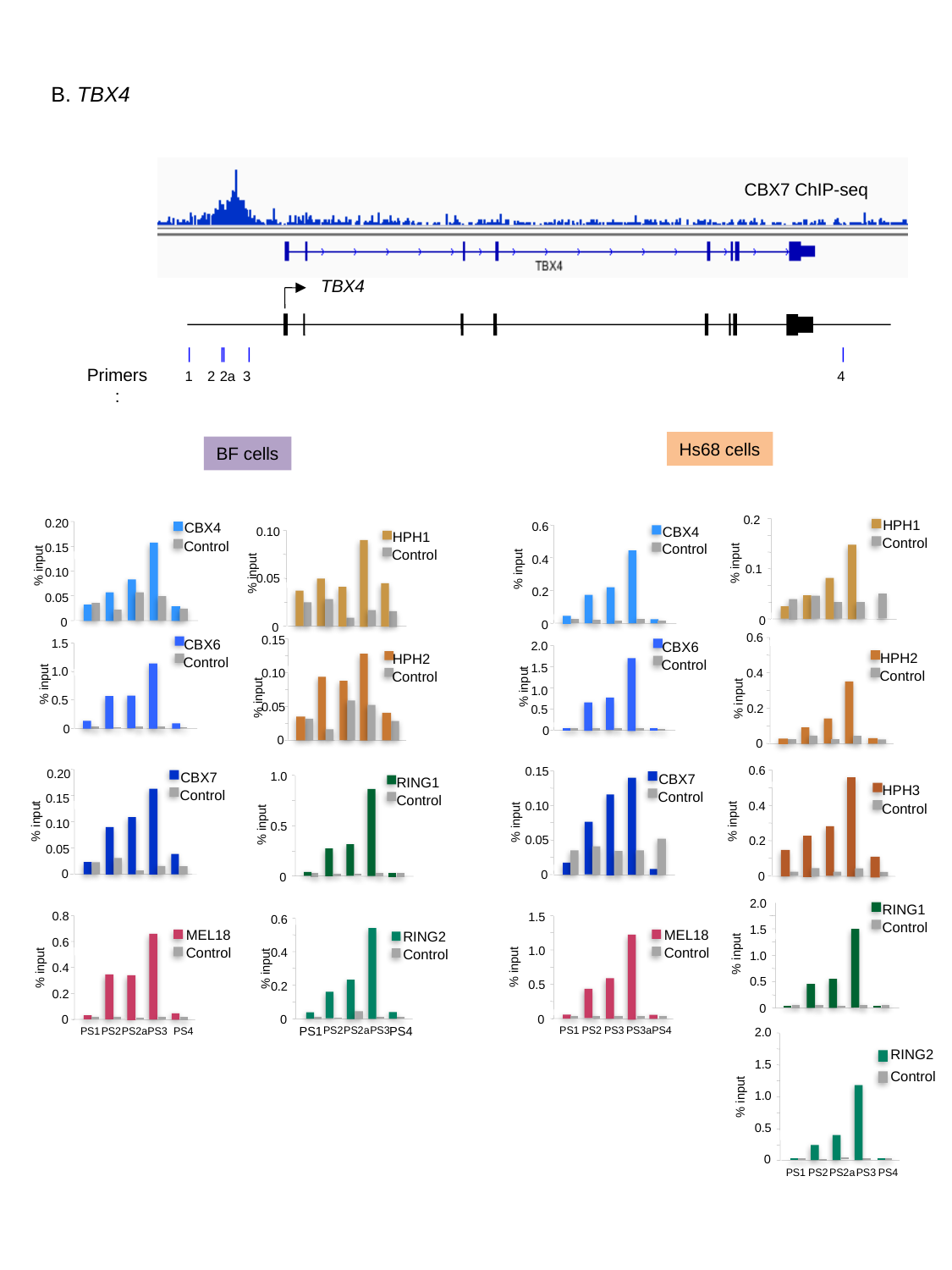

B. TBX4
CBX7 ChIP-seq
TBX4
Primers:
1
2
2a
3
4
Hs68 cells
BF cells
0.2
HPH1
Control
% input
0.1
0
0.20
CBX4
Control
0.15
% input
0.10
0.05
0
CBX6
1.5
Control
1.0
% input
0.5
0
0.20
CBX7
Control
0.15
% input
0.10
0.05
0
0.8
MEL18
0.6
Control
0.4
% input
0.2
0
PS1
PS2
PS2a
PS3
PS4
0.6
CBX4
Control
0.4
% input
0.2
0
CBX6
2.0
Control
1.5
% input
1.0
0.5
0
0.15
CBX7
Control
0.10
% input
0.05
0
1.5
MEL18
1.0
Control
% input
0.5
0
PS1
PS2
PS3
PS3a
PS4
0.10
HPH1
Control
% input
0.05
0
0.15
HPH2
0.10
Control
% input
0.05
0
1.0
RING1
Control
% input
0.5
0
0.6
RING2
0.4
Control
% input
0.2
0
PS3
PS1
PS2
PS2a
PS4
0.6
0.4
0.2
0
HPH2
Control
% input
0.6
HPH3
0.4
Control
% input
0.2
0
2.0
RING1
Control
1.5
% input
1.0
0.5
0
2.0
RING2
1.5
Control
1.0
% input
0.5
0
PS1
PS2
PS2a
PS3
PS4

## Slide 3
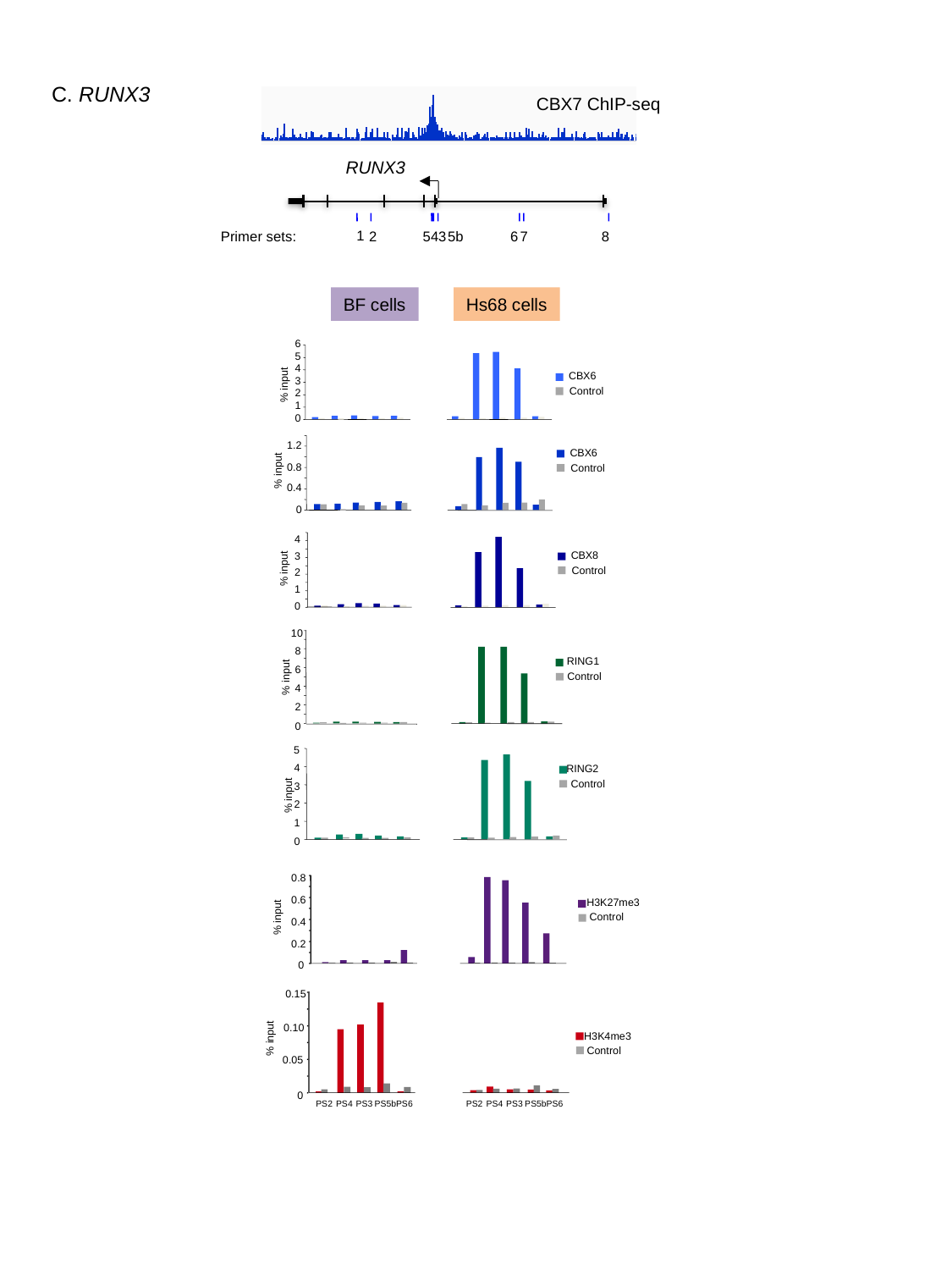

C. RUNX3
CBX7 ChIP-seq
RUNX3
1
2
5
4
3
5b
6
7
8
Primer sets:
BF cells
Hs68 cells
6
5
4
CBX6
3
% input
Control
2
1
0
1.2
CBX6
0.8
Control
% input
0.4
0
4
CBX8
3
% input
Control
2
1
0
10
8
RING1
6
Control
% input
4
2
0
5
4
RING2
Control
3
% input
2
1
0
0.8
0.6
H3K27me3
Control
% input
0.4
0.2
0
0.15
0.10
H3K4me3
% input
Control
0.05
0
PS2
PS4
PS3
PS5b
PS6
PS2
PS4
PS3
PS5b
PS6
