# Supplementary material for: Genome-wide co-localization of Polycomb orthologs and their effects on gene expression in human fibroblasts
Source: Genome Biol. 2014 Feb 3;15(2):R23. doi: 10.1186/gb-2014-15-2-r23 (PMC4053772; doi:10.1186/gb-2014-15-2-r23)
Supplement: Additional file 5: Figure S4 — ChIP-seq and RNA-sequencing profiles of the HOX clusters in BF and Hs68 cells. Upper tracks in each figure show the profiles of DNA sequence tag densities following ChIP-seq with antibodies against CBX6, CBX7, CBX8, H3K27me3 and H3K4me3 in the BF and Hs68 strains of HDF as indicated. The lower tracks show duplicate RNA-sequencing data for the corresponding genomic regions in either BF or Hs68 cells. The tag densities were normalized to the same maximum (numbers on left). [file gb-2014-15-2-r23-S5.pptx]

## Slide 1
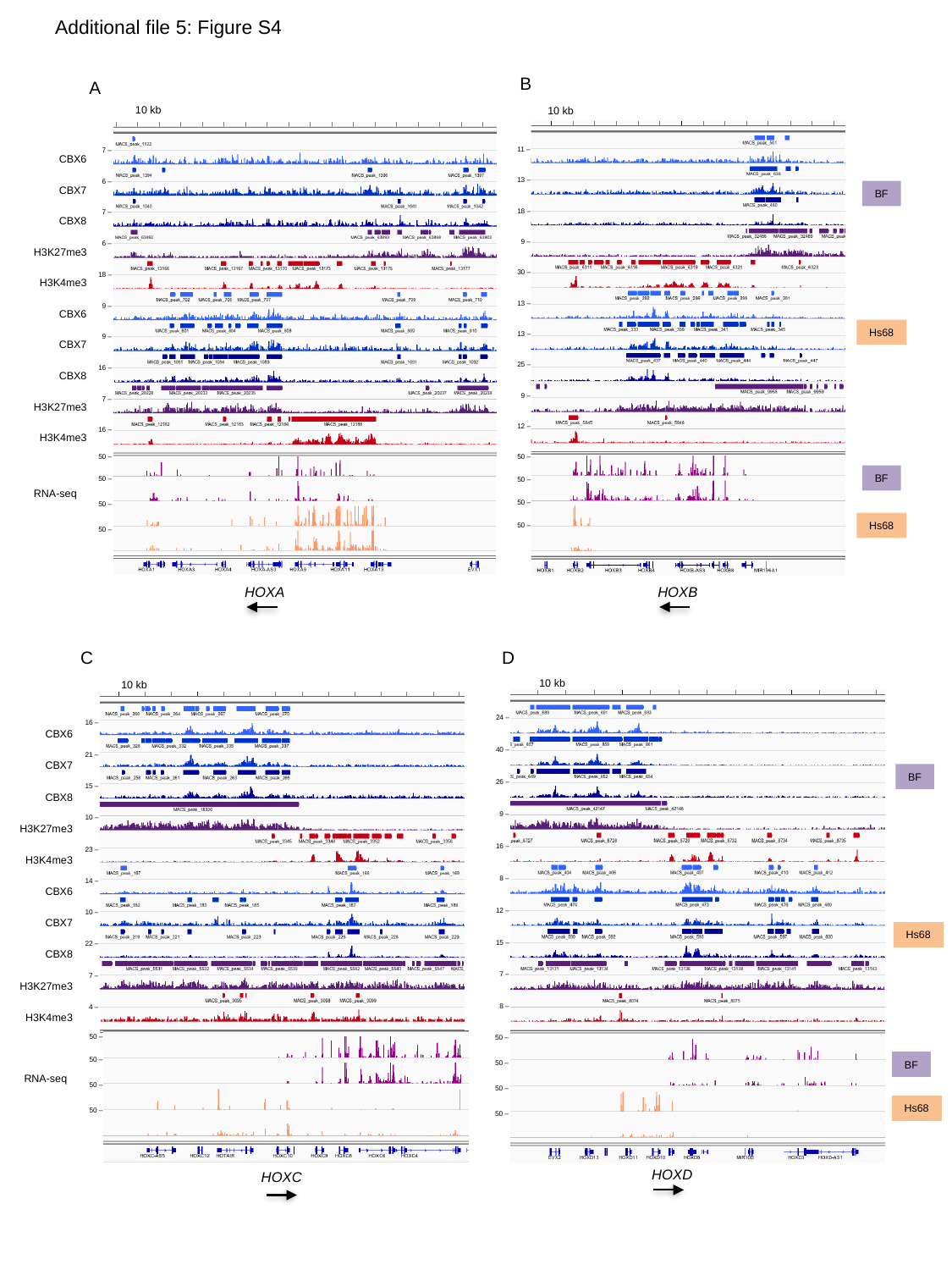

Additional file 5: Figure S4
B
A
10 kb
10 kb
11 –
7 –
CBX6
13 –
6 –
CBX7
BF
10 kb
18 –
7 –
CBX8
9 –
6 –
H3K27me3
30 –
18 –
H3K4me3
13 –
9 –
CBX6
Hs68
13 –
9 –
CBX7
25 –
16 –
CBX8
9 –
7 –
H3K27me3
12 –
16 –
H3K4me3
50 –
50 –
BF
50 –
50 –
RNA-seq
50 –
50 –
Hs68
50 –
50 –
HOXA
HOXB
C
D
10 kb
10 kb
24 –
16 –
CBX6
40 –
21 –
CBX7
BF
26 –
15 –
CBX8
9 –
10 –
H3K27me3
16 –
23 –
H3K4me3
8 –
14 –
CBX6
12 –
10 –
CBX7
Hs68
15 –
22 –
CBX8
7 –
7 –
H3K27me3
8 –
4 –
H3K4me3
50 –
50 –
50 –
BF
50 –
RNA-seq
50 –
50 –
Hs68
50 –
50 –
HOXD
HOXC
