# Supplementary material for: Genome-wide co-localization of Polycomb orthologs and their effects on gene expression in human fibroblasts
Source: Genome Biol. 2014 Feb 3;15(2):R23. doi: 10.1186/gb-2014-15-2-r23 (PMC4053772; doi:10.1186/gb-2014-15-2-r23)
Supplement: Additional file 6: Table S2 — List of antibodies used for ChIP. Table S3. List of oligonucleotide primers for PCR analysis of immunoprecipitated chromatin. Table S4. List of oligonucleotide primers used to assess RNA levels by reverse transcription and quantitative PCR. [file gb-2014-15-2-r23-S6.docx]

**Additional file 6: Table S2**. List of antibodies used for ChIP**.**

| **Protein** | **Antibody** | **Type** | **Source** |
| --- | --- | --- | --- |
| CBX4 | MRO17 | Rabbit polyclonal | In house |
| CBX6 | MRO19 | Rabbit polyclonal | In house |
| CBX7 | AB21873 | Rabbit polyclonal | Abcam |
| CBX7 | HCHA1 | Rabbit polyclonal | In house |
| CBX8 | A300-882a | Rabbit polyclonal | Bethyl |
| CBX8 | GM-LAST | Rabbit polyclonal | In house |
| MEL18 | sc1074 | Rabbit polyclonal | Santa Cruz |
| BMI1 | 05-637 (F6) | Mouse monoclonal | Millipore |
| HPH1 | H00001911-D01P | Rabbit polyclonal | Abnova |
| HPH2 | 1615 | Rabbit polyclonal | In house |
| HPH3 | MRO27 | Rabbit polyclonal | In house |
| RING1 | 2820 | Rabbit polyclonal | Cell Signalling |
| RING2 | MAb3-3 | Mouse monoclonal | Haruhiko Koseki |
| H3K27me3 | 07-449 | Rabbit polyclonal | Millipore |
| H3K4me3 | AB1012-100 | Mouse monoclonal | Abcam |
| H3 | AB1791 | Rabbit polyconal | Abcam |
| Control | AB46540 | Rabbit IgG | Abcam |
| Control | AB18413 | Mouse IgG | Abcam |

**Additional file 6: Table S3.** List of oligonucleotide primers for PCR analysis of immunoprecipitated chromatin

| **Name** | **Forward primer** | **Reverse primer** |
| --- | --- | --- |
|  |  |  |
| BMP4-PS1 | TGCATTTCATCTGCACCCCCACC | TCAATAGCCTGTCTGCGAGCCCT |
| BMP4-PS2 | GGAACCCAAGGAACCTCGCCTCTA | CATAGGACACGACACCGCAGGAAC |
| BMP4-PS3 | TGAAACACAACTCCCCCTTCCCCT | AAAACCCTCGGCCCCAAGGAATC |
| BMP4-PS4 | TCTGATTAGCGCTCGCTGGTCCT | TTCCCCTGCAGACTCCACGCTT |
|  |  |  |
| CCND2-PS1a | TGGAGTGCAACCTGGGGTTTCAT | AGGCTCCTGGAACAAGCCTCCTC |
| CCND2-PS1c | GCTATTGAAAAGCATTCTGCACT | TGCCAAACATATTGTTCTGTGAC |
| CCND2-PS2b | ACTGTCTGAAATGAAGGTGAAGC | GATTTGATGGACACTTGGTTTGT |
| CCND2-PS2c | ATTTTCAGACACCCCTAGCTTTC | GGGATGTTTGGGAGAGAAATACT |
| CCND2-PS2d | TTCACTCAGGCTAATCATGACAGT | GACCCAAGAGACTTGGTTTTGTT |
| CCND2-PS4 | ATTTCGCCTTGATGGGTGCTGGG | TTGTCCCCACAGGGCATGCTAAC |
| CCND2-PS5 | TAGTATGCAGAGGTATCCCCAAA | GCTTTTGAGTACTTGCTTCGTGT |
|  |  |  |
| CDKN2A-PS1 | GGAACCTAGATCGCCGATGTA | TGTTTTACGCGTGGAATGCAC |
| CDKN2A-PS2 | GTGGGTCCCAGTCTGCAGTTA | CCTTTGGCACCAGAGGTGAG |
| CDKN2A-PS3 | GGAGCGATGTGATCCGTTATC | TGAAATCCCAATCGTCTTCCAC |
| CDKN2A-PS5 | CTCAAAGCGGATAATTCAAGAGC | AAGCCTTAAGAACAGTGCCACAC |
| CDKN2A-PS6 | ACCCCGATTCAATTTGGCAG | AAAAAGAAATCCGCCCCCG |
| CDKN2A-PS7 | AGAGGGTCTGCAGCGG | TCGAAGCGCTACCTGATTCC |
| CDKN2A-PS9 | CAAGCTTCCTTTCCGTCATGC | GCCAGAGAGAACAGAATGGTCAGAGCCA |
| CDKN2A-PS10 | ATCGCTGAGCGATGAAGGTAG | ATCACAAAAAGGAAAGGCAAG |
| CDKN2A-PS11 | TAGGAGGCCCCATTAAGCATAC | TGTAGTTGCCAGGAGTTGGAGG |
|  |  |  |
| DLX1-PS1 | GCTGATTAGTGGGCGGTCTA | CTCTCGGTTTCCAGAACAGC |
| DLX1-PS2 | CCAACCACTCTGCCTGAAAT | ACATCGGTGCTGTCGTTACA |
| DLX1-PS3 | TGTCTCCTTCTCCCATGTCC | TGCTGACCGAGTTGACGTAG |
| DLX1-PS4 | CGTATTAACAACGGGCCCTA | AATCGTCCTGGGTTTACGG |
| DLX1-PS5 | GCCCCTCTATCAATCAGTGC | CAGGAACCTGCCTTTCTCAC |
| DLX1-PS9 | AGCAGCTATGACCTGGGCTA | TAGACCGACTCGGCACTCTT |
|  |  |  |
| GATA6-PS1 | CGATCACGGAAAGACACCTT | ATTTTCCCTCCCTGCTTCTC |
| GATA6-PS2 | GGAAATCAAATGCCCACAAC | GAATGCCTTGCTTTTGGTTC |
| GATA6-PS2a | GCCTCTCCATTCCAGAGTTTT | TCCAGAAACCGTTCTCATCC |
| GATA6-PS3 | GCATCTGCTATTGGGGAAAA | TCCCAAACTTACTCCCATCG |
|  |  |  |
| MEIS1-PS1 | GGAGCGCTTTTATGCTCAGT | TTAACGTCTCCAGCAACGTG |
| MEIS1-PS2 | CTACTCTGGACCAAGGAGCATC | CATGTCCCCCAAATTGACTT |
| MEIS1-PS3 | GTTTCCTTGGCGACTGTGTT | GGGAATCATTATGCGGTGAC |
| MEIS1-PS4 | TTCTCTGGAAGGCAGATTATCC | TCCATTCCAGTCTGAGCATTT |
| MEIS1-PS5 | GGCACTGTCACTTTCCTTCTG | GGCACTGTCACTTTCCTTCTG |
|  |  |  |
| NRN1-PS1 | TACCAAGCCACAGATGCAAG | TCATATAGATTACGAGAGGAAGGAGAT |
| NRN1-PS2 | GCAGGTTATTAATAGAATGGACAACA | AGAATGCCACAGCCTGAACT |
| NRN1-PS3 | TTGAACGAGCTCTATCAGAACAA | GAGTTCACCTTACAGCGAACAT |
| NRN1-PS4 | TTGGAATAGGCACTCTCCTTG | ACTGGACCTGGCTATGAACTACTAC |
| NRN1-PS5 | TGCATCCGTCCAAGTTCA | CCTTGTGATCTATACCGTTGGATA |
| NRN1-PS6 | GATTATTATCGCGCCGTCTT | ACCGGTAACATCAACCAACATA |
| NRN1-PS7 | CCTGGCTCTCACTGGCTAA | TGCCAGTTCACAATCACTCAC |
|  |  |  |
| RUNX3-PS2 | GGGGGCCTTTGCACTGCTCTTTT | GGAAGGCTGTGTGTGTGCCACTT |
| RUNX3-PS3 | TCAAAAGGCATCCGCCTCTCCGT | AAGGATGCACCTGCCGGGAATTG |
| RUNX3-PS4 | TGCAGAGGCCAATAAACTGGGCC | AGGGCTGCTACCCCCACTCAATT |
| RUNX3-PS5 | AATTGAGTGGGGGTAGCAGCCCT | ATGATGTGGGGTGCAGTTCCCCT |
| RUNX3-PS5 | TGAAGCCCCATGCCAAAGAGCTG | TGGCCCCTTGTCTGAATGCGTTG |
|  |  |  |
| TBX2-PS1 | CAGGACAATCTGGTTATTCATCTG | GCTTATCTGGTCTCTCTTATCCTCA |
| TBX2-PS2 | GCCAGAGAGGAGGTACTAAGAGG | CAACTCCTCATCACCGCATA |
| TBX2-PS3 | CACACACACACACCATCACTTC | GTCTGCGAGCACCTGTGA |
| TBX2-PS3a | GAGCCAACGACATCCTGAA | CGCACCTTGTCATTCTGGTA |
| TBX2-PS4 | ATGGTGAAGGTGGAATGGAC | CTCTCTCTCTCGCTCCTTGC |
|  |  |  |
| TBX4-PS1 | TACTGTGCTGACTGGCATCC | ATGAATGACCTGCAAGCTGA |
| TBX4-PS2 | CTCTGAGTAACGCGATGAATTG | CTGTCACGGCCTTGTTCTTAG |
| TBX4-PS2a | AGAATATGCAAGGCCAAGGA | AAGGCTCTGAGTCTAGTGTTCTGAT |
| TBX4-PS3 | TTGTTCACTATGTCACCTCGATATT | TTCCTGTCACTGTTCCATCATT |
| TBX4-PS4 | TGTGTATGCGCTGTGCTG | TGAGGTTCGAGGCACATTC |

**Additional file 6: Table S4**. List of oligonucleotide primers used to assess RNA levels by qRT-PCR

| **Gene** | **Sense** | **Antisense** |
| --- | --- | --- |
|  |  |  |
| *ATF3*  *BMP4* | GTTTGCCATCCAGAACAAGC  TCCACAGCACTGGTCTTGAG | TCATCTTCTTCAGGGGGTACC  TGGGATGTTCTCCAGATGTTCT |
| *CBX7* | CCTCCCCATCCAACCTAAAT | GAGGTCCCTCCACAGGAATAC |
| *CDKN2A* | CGGTCGGAGGCCGATCCAG | GCGCCGTGGAGCAGCAGCAGCT |
| *DKK2* | GGCAGTAAGAAGGGCAAAAA | CCTCCCAACTTCACACTCCT |
| *EN1* | GCACACGTTATTCGGATCG | GCTTGTCCTCCTTCTCGTTC |
| *FGF10* | GAAGAACGGGAAGGTCAGC | CCCCTTCTTGTTCATGGCTA |
| *FOXE1* | CGTCCTGCTCAAAAGTTCAA | AACCTAAAGTCCCAGGATTGG |
| *GAD1* | TGACTTCTCTAATCTGTTTGCTA | CATAGTTGAGGAGTATGTCCACC |
| *GAS1* | TCTCGACAGCTGTTCATTTCC | GCAGAAGGTCCCCTTTCG |
| *GATA2* | GGCTCGTTCCTGTTCAGAA | GGCATTGCACAGGTAGTG |
| *GDF6* | TGCCAGCTTTTTCCAGTCTT | CCCACCAGCTCTTCTTTGTC |
| *IGFBP2* | AAGGGTGGCAAGCATCAC | CTGGTCCAGTTCCTGTTGG |
| *IGFBP5* | GGTTTGCCTCAACGAAAAGA | AGATCTTGGGGGAGTAGGTCTC |
| *ISL2* | GACGGGAAGACCTACTGCAA | AGCACTCGATGTGGTACACG |
| *MEIS1* | CACAAAAAGCGTGGCATCT | GATGGTGAGTCCCGTGTCTT |
| *NKX6-1* | CGTTGGGGATGACAGAGAG | CGAGTCCTGCTTCTTCTTGG |
| *NRN1* | ACGACAAGACGAACATCAAGAC | TCAGTTTATCCCACATATCTTTCG |
| *RPS17* | GCACCAAAACCGTGAAGAAG | TCTTGTTGCGGAGCTTTTTG |
| *RUNX3* | GCAGTGGGCGAGGGAAGAGTTTCA | GCTTGGTCTGGTCCTCCAGCTTCT |
| *SFRP2* | AGGACAACGACCTTTGCATC | TTTTTGCAGGCTTCACATACC |
| *TBX2* | GACAAGCACGGCTTCACC | GTTGGCTCGCACTATGTGG |
| *TBX4* | CCATCGCTACAAGTTCTGTGAC | GAATCCGGGTGGACATACAG |
| *TGFB2* | CCAAAGGGTACAATGCCAAC | CAGATGCTTCTGGATTTATGGTATT |
| *VCAN* | GCACCTGTGTGCCAGGATA | CAGGGATTAGAGTGACATTCATCA |
